# Use of industrial hemp byproducts in ruminants: a review of the nutritional profile, animal response, constraints, and global regulatory environment

**DOI:** 10.1186/s42238-025-00279-7

**Published:** 2025-05-14

**Authors:** Agung Irawan, Hunter Buffington, Serkan Ates, Massimo Bionaz

**Affiliations:** 1 Oregon State University, Corvallis, OR 97331 USA; 2 Agriculture Policy Solutions, Loveland, CO 80538 USA; 3 Universitas Sebelas Maret, Surakarta, 57126 Indonesia

**Keywords:** Cannabis sativa, Feed alternative, Hemp byproduct, Industrial hemp, Ruminants

## Abstract

**Background:**

The legalization of industrial hemp, *Cannabis sativa* L., which contains < 0.3% ∆^9^-tetrahydrocannabinol (∆^9^-THC), in many countries, has led to a significant rise in its cultivation. Consequently, byproducts derived from industrial hemp processing have resulted in numerous emerging potential feed ingredients, including hempseed byproduct (HSB; hempseed cake or hempseed meal) from seed processing, hemp hurds, and hemp stalk from fiber processing, and spent hemp biomass (SHB) from cannabinoids extraction. Research to assess the potential use of these byproducts as animal feed is progressing.

**Method:**

We provide an overview of the nutritional characteristics of the various hemp byproducts and provide a meta-analysis of 26 empirical studies investigating the use of hemp byproducts on ruminants. Using those studies, we delved into a comprehensive assessment regarding the effects of HSB and SHB on the health and performance of the animals.

**Results:**

Overall, HSB and SHB possess excellent nutritional profiles due to their high protein content and, particularly for HSB, desirable fatty acids profile can partially replace protein-source ingredients such as soybean meal, dried distillers’ grains with soluble, canola meal, and alfalfa in the diets of ruminants. These byproducts contain diverse phytochemicals with antioxidants, anti-inflammatory, and antimicrobial properties. Data do not reveal any significant concern for the health of the animals fed hemp byproducts and, with few exceptions, the data do not indicate a substantial effect on performance; dietary inclusion of HSB, however, has a deleterious impact on rumen fermentation and nutrient digestibility when given as raw HSB without dehulling, reducing the growth performance of meat-producing ruminants. On the other hand, SHB has low palatability overall but does not impair production performance.

**Conclusions:**

Although they can be promising feed ingredients for ruminants, their present use as feed ingredients is limited by the residuals of THC and CBD. Our comprehensive review of the current legal status of hemp byproducts worldwide highlighted a complex scenario with some countries allowing the use of hemp byproducts as feed ingredients, some with no clear regulations, and some countries where a path for the regulation has started, such as the US. Still, no hemp byproducts are yet legal as a feed ingredient for ruminants.

**Supplementary Information:**

The online version contains supplementary material available at 10.1186/s42238-025-00279-7.

## Introduction

Hemp (*Cannabis sativa* L.) is considered one of the most versatile crops, with more than 25,000 diversified uses (Frontier 2022). In the US, hemp became illegal after the Second World War until the 2018 Farm Bill legalized it. Industrial hemp is defined as cannabis with < 0.3% of the psychoactive ∆^9^-tetrahydrocannabinol (∆^9^-THC). Hemp is a fast-growing industry with an estimated value of $3.7 billion globally in 2018 (Frontier 2022). A 17.5% annual growth rate is expected until 2030 (Market Analysis Report 2022). Historically, the U.S. has been a leading country in the consumption of hemp products, with an exponential increase in the import of hemp oil and seed in the last twenty years (Cherney and Small 2016). In 2018, the U.S. was the second biggest hemp market, with $1 billion in sales (Frontier, 2022).

Large quantities of industrial hemp by-products are produced from at least three main hemp uses: fiber, cannabinoid, and oil extraction. The primary byproducts from these processing industries include hempseed meal (a.k.a. hempseed cake), which is produced during the extraction of oil from the seeds, and hemp biomass remaining after extracting cannabinoids, predominantly cannabidiol (a.k.a spent hemp biomass or SHB), both of which can be utilized as animal feed. Hempseed meal is used interchangeably with hempseed cake, as they are technically derived from the same process (Ely and Fike 2022). Therefore, the term “hempseed byproduct” will be employed in this article to encompass both hempseed meal and hempseed cake (i.e., hempseed byproducts or HSB). The hemp plant is not intentionally cultivated for animal feed (Kolodziejczyk et al. 2012). However, its byproducts have received special attention in the last few years to exploit their potential as feed ingredients for livestock and poultry due to their nutritional values (Altman et al. 2024; Shariatmadari 2023). Applying high-pressure and low-temperature processing (Aluko 2017) is especially advantageous in preserving the nutritional components of exhausted byproducts (Parker et al. 2022; Vonapartis et al. 2015). The byproducts from hemp contain high concentrations of protein and functional fatty acids, especially polyunsaturated fatty acids, including omega-6 in the form of C18:2 n-6 and omega-3 in the form of C18:3 n-3 (Callaway 2004; Mattila et al. 2018; Silversides and Lefrançois 2005). Hemp also contains many other bioactive compounds that can positively affect health, as demonstrated in monogastric animal studies (Aluko 2017).

Studies previously reviewed (Semwogerere et al. 2020) and recent data from Parker et al. (2022) revealed that byproducts from the hemp industry could be high-quality feed ingredients for livestock. However, the possibility of residuals of ∆^9^-THC and cannabidiol (CBD) in animal products is the primary concern for regulatory agencies around the world, including the US Federal Drug Administration Center for Veterinary Medicine (FDA-CMV) and the Association of American Feed Control Officials (AAFCO) (2018). This is an apparent contradiction, as the use of CBD is legal in humans, as well as the consumption of hemp seed, which contains excellent properties for human health (Farinon et al. 2020). In addition, CBD can be of great interest to the livestock industry due to its potential benefits for the presence of antioxidants, antimicrobials, and anti-inflammatory compounds (Fiorini et al. 2020).

A recently published review article (Altman et al. 2024) broadly discussed industrial hemp byproducts as feed for livestock. To add to the literature, our review attempts to systematically summarize the impacts of dietary hemp byproducts on ruminants using a meta-analysis approach and considering additional published studies, especially six relevant in vivo studies that were recent or not previously considered (Addo et al. 2023b; Addo 2022; Irawan et al. 2024; Karlsson et al. 2012; Karlsson et al. 2010; Semwogerere et al. 2023a). We additionally discuss essential aspects of industrial hemp as a ruminant feed ingredient, including global industrial hemp production, global regulatory environment, nutritional characteristics, and impact on animals’ production and performance that are not covered in the previously published review.

### Global hemp production and utilization

**Fig. 1:**
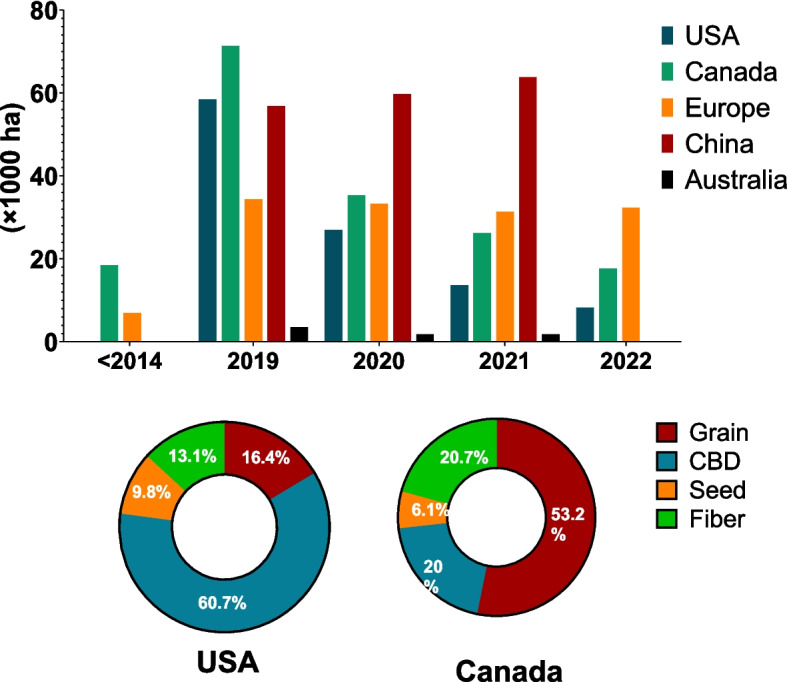
Statistics of industrial hemp cultivation and type of industrial hemp in various regions.^.^ Data were analyzed from Brightfield Group (Team, 2021); Canada Hemp Licensing Statistics (Canada, 2022); National Hemp Report (National Hemp Report, 2023); USDA Crop Acreage Data (Farm Service Agency and U.S. Department of Agriculture, 2023)

Global hemp cultivation increased rapidly from 2014 until 2019, reaching the record following the 2018 Farm Bill implementation (National Agricultural Statistics Service (NASS) 2022). China, Canada, Europe (mainly France), and the USA are among the top global hemp producers in the world (Frontier 2022). As shown in Fig. 1, Canada was the leading producer in 2019, but China took the lead in the following two years (2020–2021). The type of hemp cultivated in different regions is quite distinct. In contrast, approximately three-quarters of hemp in China is intended for textiles, and the rest is for cosmetics, CBD, and food (Victoria, 2022), while > 60% of hemp grown in the US is processed for CBD production. 53% of hemp in Canada is cultivated to produce grain, 20% for fiber, and 21% for CBD (Canada 2022). In Europe, hemp is mainly grown for seed, fiber, or as a dual-purpose (fiber and seed) (Wylie et al. 2021), with an increase in the cultivation of hemp for seed as the demand for hemp seed for human consumption is booming in the European market (Michael Carus and Luis Sarmento 2016). Similarly, most hemp is grown in Australia for seed production (Victoria 2022).

In most European countries and Great Britain, hemp varieties with < 0.3% ∆^9^-THC can grow (European Union 2021). In Canada and Australia, hemp cultivation was allowed in 1998 (Cherney and Small 2016). The official regulation about the cultivation of hemp in China is not available. The US is the most recent country to allow the cultivation of industrial hemp (McMinimy 2019). Research institutes and universities in the US were authorized to grow hemp for research purposes under the 2014 Farm Bill pilot program (Mark et al. 2020). Then, starting in 2018, hemp with ≤ 0.3% ∆^9^-THC was allowed to be cultivated in the US after the 2018 Farm Bill (Johnson 2019). This legal status has prompted a surge in cultivation, from 10,000 hectares in 2017 to 60,000 hectares in 2019 (Mark et al. 2020). Due to this increase in hemp cultivation in the US, 40 universities established research programs on hemp-related topics, including the Global Hemp Innovation Center at Oregon State University, established in June 2019 as the largest hemp research consortium (Steiner 2021).

The US has the highest CBD economic value (Frontier, 2022), primarily due to production in five states: Utah, Kentucky, Oregon, Colorado, and California. The 2021 market size of the hemp industry (i.e., industrial hemp value) was $824 million, with 13,550 hectares harvested and 80% utilized. Hemp intended for CBD production contributed 36% of the value (National Agricultural Statistics Service (NASS) 2022). According to some predictions, the market size of extracted CBD is expected to grow continuously, reaching up to $12 billion in 2026 (Team 2021). However, according to the 2022 National Hemp Report from the USDA (National Agricultural Statistics Service (NASS) 2022), there was a downward trend in hemp value in the US ($238 million or 29% compared to 2021) due to a decrease in cultivation (7,386 hectares or 54.5% compared to 2021) and production (from 1.38 tons/hectare in 2021 to 1.07 tons/hectare in 2022). Hemp for all types of utilization decreased, but the most significant decrease in production was observed in hemp for cannabinoid extraction (34% in 2022 vs. 2021), with grain (56%) and fiber (63%) having a less dramatic decrease in production from 2021 to 2022. The most considerable reduction in hemp cultivation for cannabinoid extraction was mainly due to the 2019 surplus production that has not yet been depleted (Forbes 2022).

The SHB obtained after the extraction of CBD (or other cannabinoids) is treated mainly as waste material that goes to composting or landfilling. Although hemp byproducts possess favorable nutritional qualities suitable for feed ingredients, they are not yet legalized as feed ingredients for animals under the US federal law and European Union regulation (AAFCO 2018; EFSA 2011). Despite any hemp products being illegal as feed ingredients for animals, hulled hemp seeds, hemp seed protein, and hemp seed oil are categorized as generally recognized as safe (“GRAS”) by the FDA, and therefore, they can be marketed as a food ingredient for humans (FDA 2018a; FDA 2018b; FDA 2018c). Given the current regulatory framework, it is critical to thoroughly assess the safety of hemp byproducts across several animal species. A letter was issued in 2018 by AAFCO to call for action by researchers to conduct thorough studies to assess (1) the impact of SHB on the health and safety aspects of animals, (2) the impact on safety of the food derived from animal origin; (3) risk on farmers, ranchers, and feed manufacturers especially when involving interstate trading activity; and (4) impacts on interstate and international commerce and market access (AAFCO 2018).

### Hemp processing

Hempseed mainly undergoes a milling process to produce flour for food ingredients or oil by pressing or solvent extraction. Approximately 25–35% of hempseed is oil, 20–30% is protein, and the rest are carbohydrates and fiber (Ely and Fike 2022). Hempseed is harvested for flour and oil and sometimes for both (i.e., dual-purpose production). Flour production often involves dehulling and defatting processes to produce protein concentrate and hemp oil, leaving hempseed hull as the main byproduct. Hempseed is frequently processed exclusively to produce hemp oil that provides hempseed meal or hempseed cake as the main byproduct (Burton et al. 2022).

Hemp biomass, comprising flowers and leaves, or the entire plant, is extracted to produce cannabinoid oils, particularly CBD. Cannabinoids in hemp are produced in glandular trichomes enriched in the flower (Flores-Sanchez and Verpoorte 2008). Cannabis is mostly a dioecious species, and CBD is extracted from female hemp that contains the flowers. In the last few years, efforts have been made to optimize the extraction process to obtain higher yield and purity of cannabinoids using various operating processes (Moreno et al. 2020). Hydro-distillation is a traditional method for cannabinoid extraction in hemp (Al Ubeed et al. 2022). Several other extraction methods exist, including Soxhlet extraction, accelerated solvent extraction, microwave-assisted extraction, and ultrasound-assisted extraction (Al Ubeed et al. 2022). These extraction methods use organic solvents such as chloroform, ethyl acetate, n-hexane, methanol, ethanol, and acetone (Valizadehderakhshan et al. 2021). In most extraction methods, the flowers undergo trimming and drying at low temperatures to prevent the conversion of acid phytocannabinoids to bioactive neutral phytocannabinoids (Valizadehderakhshan et al. 2021). Decarboxylation starts to become substantial at a temperature > 80℃ (Wang et al. 2016). The dried flower is then milled into a small particle size and immersed in the extraction tank containing a specific solvent such as CO_2_ or ethanol.

In many reports, they can extract phytochemicals in hemp with recovery ranging from 60 to 99% but require relatively long-time extraction, up to 24 h (Qamar et al. 2021). Moreover, waste generated from the extraction process could pose environmental hazards (Qamar et al. 2021) and require significant energy consumption. Hence, prioritizing environmentally friendly extraction processes has become a focal point in cannabinoid research. New extraction technologies have been developed recently, including microwave-assisted extraction (MAE), supercritical fluid extraction (SFE), and pressurized liquid–liquid extraction (PLE) that are more efficient and have a higher selective ability to extract phytocannabinoid compounds (Qamar et al. 2021).

#### Microwave-assisted extraction

It works by increasing radiation temperature to release volatile organic compounds (VOC) via an azeotropic distillation process (Fiorini et al. 2020). This process helps with faster diffusion of the target VOC from the material and thus saves energy, time, and cost. Fiorini et al. (2020) demonstrated a reduced time of CBD extraction from 240 min using hydro distillation to 115 min with MAE with higher CBD yield (5.6 vs 9.3%, respectively).

#### Supercritical fluid extraction

Utilizing carbon dioxide (CO_2_) at low temperatures (50–60 °C) helps prevent thermal degradation of the phytochemicals (Kitrytė et al. 2018). The CO_2_ used as the solvent can be recycled and reused without requiring toxic solvents in the operation. In its supercritical form, CO_2_ is highly effective in extracting cannabinoids owing to its liquid-like solvating power and gas-like mass-transferring ability inside the substance, thus increasing the yield of extracted cannabinoids. In the literature, highly variable results were reported due to several factors that significantly influence the yield and purity outputs of the cannabinoids, including pre-treatment conditions, level of solvent, temperature, flow rate, and pressure (Qamar et al. 2021). Compared to traditional methods, SFE resulted in higher extraction efficiency for most cannabinoid compounds, particularly CBD and THC. In addition, it was proposed that employing ethanol as a co-solvent could improve efficiency. In this regard, Rovetto and Aieta (Rovetto and Aieta 2017) demonstrated that using 40% ethanol as a co-solvent during SFE resulted in 89–92% extraction efficiency of THC from hemp flower. Another SFE experiment obtained 94–98% cannabinoid recovery, including CBD and THC, by using 5% ethanol co-solvent and increasing pressure from 300 to 500 bars, which further improved CBD recovery. Ethanol helps to enhance extraction because acidic forms of cannabinoids have a higher affinity to polar compounds such as ethanol (Ribeiro Grijó et al. 2019). However, to improve the recovery of acidic compounds such as THCA and CBDA, higher pressure is required, and this study demonstrated that the pressure of 1000 and 1300 bars increased the recovery of these acidic forms. Higher pressure SFE (> 700 bars; a.k.a. ultra-high pressure SFE) is advantageous in enhancing the solubility of organic compounds to increase extraction efficiency and decrease extraction time (Moreno et al. 2020). Notably, ∆^9^-THC is less soluble than CBD in supercritical CO_2_; therefore, higher pressure and temperature are needed to improve the extraction efficiency of THC (Perrotin-Brunel et al. 2010). SFE was also reported to extract 98% of ∆^9^-THC with 85% purity using SFE operated between 180 and 230 bars (Perrotin-Brunel et al. 2011). In addition, SFE-CO_2_ is also effective in extracting trace amounts of cannabinoids from hemp residuals, hemp fiber, industrial hemp seed, and hemp waste and, therefore, can be an alternative method for biorefinery of industrial hemp byproducts. After cannabinoid extraction, trace amounts of ∆^9^-THC (< 0.1% w/w) and CBD (< 2% w/w) are left (Parker et al. 2022). It was demonstrated that SFE-CO_2_ could recover 29% CBD of hemp dust samples from the hemp fiber industry, with CBD concentration at 0.58% dry weight (Attard et al. 2018).

#### Pressurized-liquid extraction

The PLE uses the thermo-chemical decarboxylation principle under high pressure and temperature to extract target cannabinoid compounds from complex matrixes (Olejar et al., 2021). The PLE was developed to address the carbon emissions associated with SFE while enhancing process adjustability. Compared to SFE, PLE can be performed at a lower temperature while maintaining the solvent in the liquid phase, resulting in a lower energy cost (Serna‐loaiza et al. 2020). This process increased compound solubility and reduced hindering factors such as surface tension and viscosity (Solana et al. 2015). Studies have demonstrated that the PLE process can be done between 6 and 60 min to extract > 90% of targeted cannabinoids (Olejar et al., 2021; Olejar and Kinney 2021; Serna‐loaiza et al. 2020).

#### Enzyme-assisted extraction (EAE)

Enzymes such as cellulose, pectinase, or their combination help increase cannabinoid extraction recovery by releasing the phytochemicals from the cells of plants (Al Ubeed et al. 2022). An increase of 20% in cannabinoid recovery was reported by the application of EAE (Latif and Anwar 2009). Currently, few studies report the application of EAE for cannabinoid extraction, primarily due to the high operational costs involved (Al Ubeed et al. 2022).

#### Multistep biorefinery process

With the combination of SFE-CO_2_, PLE, and EAE, > 93% CBD and CBDA were recovered from threshing residues of hemp containing 0.01–0.02% CBD and 0.03–0.23% CBDA (Kitrytė et al. 2018). The authors optimized the SFE-CO_2_ at 465 bars, 70 °C, and 120 min, where PLE and cellulolytic EAE contributed to 61 and 39% recovery of lipophilic compounds, including CBD and CBDA. Another work also demonstrated that PLE using ethanol at 60 bars, 100 °C, and 60 min could recover total CBD and ∆^9^-THC by 99% and 83%, respectively (Serna‐loaiza et al. 2020). The advancement of novel and more efficient technologies for cannabinoid extraction holds promise for the hemp industry and the potential utilization of its byproducts in animal feed. Highly efficient extraction methods can allow the obtaining of more phytochemicals out of hemp for pharmaceutical purposes but would also produce byproducts that do not contain detectable cannabinoid residues. However, the potential loss of phytochemicals by highly efficient extraction method means that the byproducts would no longer have nutraceutical effects for livestock.

### Nutritional characteristics of hemp and hemp byproducts

**Fig. 2:**
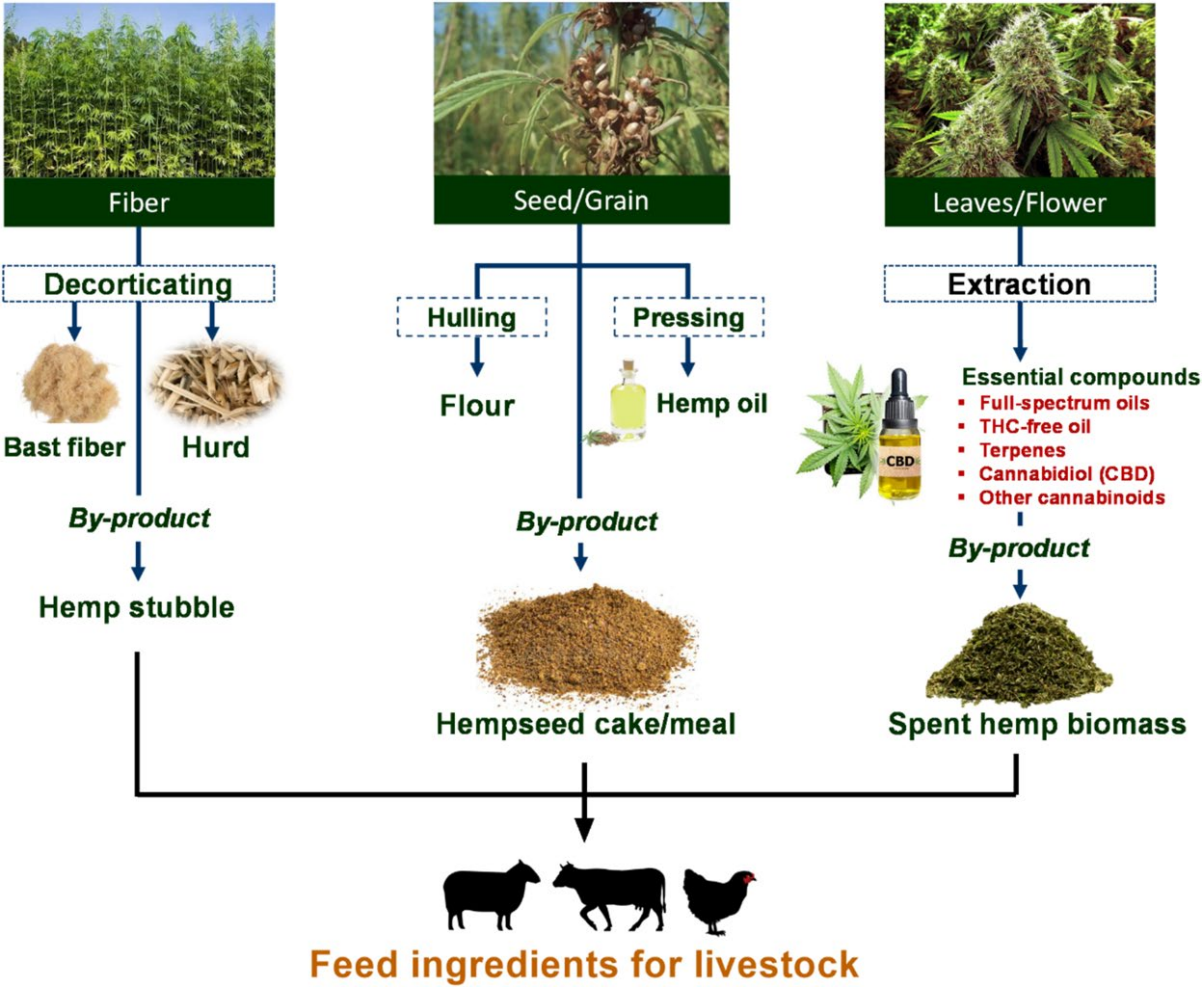
Schematic representation of different purposes of cultivating hemp and their potential byproducts as a feed for livestock

Industrial hemp is grown for fiber, seed, oil (i.e., hempseed oil or HSO), and cannabinoid production. A schematic representation of industrial hemp processing and the relative byproducts is illustrated in Fig. 2. Among byproducts of hemp utilization, HSB and SHB can be fed to livestock.

#### Proximate and micromineral composition

##### Whole plant

Reports on the nutritional composition of entire hemp plants are scant. Proximate analysis showed that the whole hemp plant has a relatively similar nutritional profile with forages commonly fed to ruminants as indicated by a moderate percentage of CP (14–19%) and fiber (NDF content of 44.5 to 46.5%) with the best nutritional profile obtained from hemp harvested during mid-vegetative stage (Peiretti 2009). The composition of fodder hemp harvested 42 days post-planting, with 17% crude protein (CP), 39% neutral detergent fiber (NDF), and 27% acid detergent fiber (ADF), is similar to forages utilized in silage production (Stringer 2018). More recently, studies in the USA and Germany reported a lower CP content of whole hemp plants harvested for seed (7–8% respectively) compared with leaves and flowers (13–21%) (Kleinhenz et al. 2020a; Wagner et al. 2022). The lower CP content of hemp plants for seed production could be attributed to the plant maturity as they are in a late vegetative stage, resulting in more fibrous plant material than hemp for CBD production, which is mainly harvested during their vegetative stages to obtain leaves and flowers. Thus, the whole plant could be considered a decent feed for herbivores, especially ruminants, if harvested during the vegetative stage; however, the nutritive quality of the plant decreases substantially as it progresses toward maturity (Stringer 2018).

##### Hempseed byproducts

Hempseed byproducts (hempseed cake or hempseed meal) are seed byproducts after oil extraction differ in form (Abrahamsen et al. 2021b; Winders et al. 2023). In Europe, extensive exploration to use hemp byproducts as animal feed is promoted by the increasing volume of hemp grown for dual-purpose production, i.e., fiber and seeds production. The most recent statistics reported that hemp seed production represented over 80% of total hemp production in Europe (Mirizzi and Troyano 2020). Among livestock’s most critical nutritional aspects, Hemp seed possesses highly desirable protein and lipid profiles. The chemical composition profiling of hemp seeds reveals similar levels of protein and lipids across different geographical regions. This is demonstrated by data obtained from the chemical analysis of over 50 hemp seed cultivars originating from Canada, China, Italy, and Spain (Alonso-Esteban et al. 2022a; Arango et al. 2024; Galasso et al. 2016; Song et al. 2022). As summarized in Table 1, hemp seeds have, on average, high CP (28.0 ± 7.0%) and fat content (28.5 ± 7.7%). Once oil is extracted from hemp seeds, the CP becomes concentrated, resulting in increased CP in HSB (37.1 ± 9.6; Table 1) compared to hemp seeds. Compared to the commonly fed byproduct for ruminants, distillers dried grains with soluble (DDGS) (Lobos et al. 2021; Winders et al. 2023), HSB has a higher CP content. However, it falls short compared to canola meal (Addo et al. 2023a) or soybean meal (Šalavardić et al. 2021). The fiber content of HSB is quite similar to that of hempseed, while dehulled hempseed has much lower fiber content (Table 1). With a few significant exceptions, HSB has a mineral composition similar to soybean meal (SBM) but contains a lower amount of Fe and a higher amount of P and Mn. Based on data gathered from existing literature, HSB appears to be a viable feed option for ruminants. However, if the seeds remain un-dehulled, the high NDF and relatively low energy content are limited. These factors should be carefully weighed when incorporating HSB into the diet to prevent potential reductions in animal production outputs.

**Table 1:** Chemical composition of various hemp and hemp byproducts

Items	Hemp and/or hemp byproduct^a^									SBM^b^	Alfalfa^c^
	Whole plant	Leaves	Flower	HS	DHS	HSB	SHB	HOR	HH		
*Chemical composition (%* ± *SD* ^d^ *)*											
CP	13.4 ± 5.1	13.0	19.8 ± 5.5	28.0 ± 7.0	27.0 ± 3.6	37.1 ± 9.6	20.2 ± 2.1	44.2	10.3	47.0	20.8
EE	2.40 ± 0.42	8.9	10.5 ± 6.5	28.5 ± 7.7	52.1 ± 1.7	9.9 ± 2.1	4.3 ± 2.3	2.16	-	9.50	1.60
NDF	53.6 ± 15.7	44.7	37.8 ± 12.8	40.5 ± 8.8	4.9 ± 1.5	39.7 ± 10.8	40.1 ± 9.7	20.1	64.9	13.6	36.9
ADF	39.3 ± 13.2	20.8	23.3 ± 4.5	47.0	-	29.5 ± 7.7	29.6 ± 7.2	9.0	-	8.0	30.8
ADL	-	-	-	11.6 ± 3.5	-	13.2 ± 1.2	12.2 ± 0.5	2.91	-		
Ash	11.4 ± 2.9	21.2	19.4 ± 5.9	6.1 ± 1.3	6.4	7.8 ± 2.2	17.7 ± 2.6	12.8	3.90	5.0	10.0
NFC	2.50	15.3	13.3 ± 9.8	16.8	-	11.8 ± 11.8	21.4 ± 11.8	1.32	-		
ME	-	-	-	1.73	-	2.50 ± 0.39	1.91 ± 0.42	3.53	-	3.58	2.39
NEL	0.81	-	1.41	-	-	1.71 ± 0.03	1.30 ± 0.25	-	-	2.40	1.36
*Minerals (%* ± *SD)*											
Ca	1.40	4.30	2.95 ± 0.65	0.30 ± 0.36	0.08 ± 0.01	0.24 ± 0.06	3.45 ± 0.57	0.26	-	0.37	1.87
P	0.30	0.40	0.75 ± 0.35	0.83 ± 0.11	1.11 ± 0.06	1.63 ± 0.62	0.78 ± 0.10	2.08	-	0.69	0.34
K	1.10	3.30	2.15 ± 0.25	0.57 ± 0.13	0.92 ± 0.10	1.58 ± 0.39	2.24 ± 0.05	-	-	2.24	3.15
Mg	0.20	0.50	0.45 ± 0.05	0.38 ± 0.02	0.70 ± 0.16	0.81 ± 0.23	0.57 ± 0.05	-	-	0.29	0.42

^a^*HS* hemp seed, *DHS* dehulled hemp seed, *HSB* hemp seed byproducts, *SHB* spent hemp biomass, *SBM* soybean meal, *HOR* hemp oil residue, *HH* hemp Hurd, *CP* crude protein, *EE* ether extract, *NDF* neutral detergent fiber, *ADF* acid detergent fiber, *ADL* acid detergent lignin, *NFC* non-fibrous carbohydrates, *ME* metabolizable energy for dairy cows, *NEL* net energy for lactation (dairy cows). Values were obtained from primary studies reported in Table 1 and (Peiretti, 2009; House et al, 2010; Pojić et al, 2014; Galasso et al, 2016; Farinon et al, 2020; Alonso-Esteban et al,^ 2022^; Song et al, 2022; Mohamed et al, 2023)

^b^Feedtable.com

^c^Parker et al. (Parker et al. 2022)

^d^If available; values without SD indicate they were obtained from one study

##### Spent hemp biomass

**Fig. 3:**
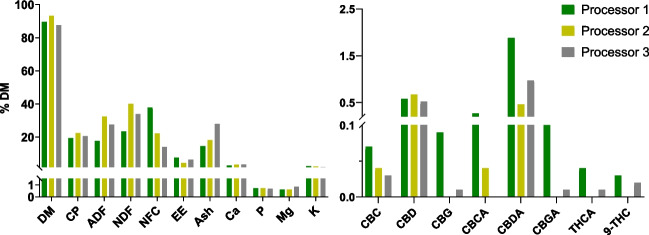
Chemical composition and cannabinoids concentration of spent hemp biomass from three different processors in Oregon, USA

Only three studies are reporting the nutritional composition of SHB, and both reported a very similar nutritional profile (Irawan et al. 2024; Parker et al. 2022; Wang et al. 2023). Oregon is among the top states in the US that processed > 90% hemp for CBD oil production, leading to large amounts of leftover biomass. Nutritional data of SHB from three different SHB processors in Oregon is available in Fig. 3. The SHB has similar nutritional characteristics to high-quality forages commonly used as ruminant feed, such as alfalfa (Parker et al. 2022; Vonapartis et al. 2015), as shown from the CP, NDF, and ADF content of SHB vs. alfalfa in Table 1. In particular, the plant is harvested at the flowering stage, and the extraction is done chiefly using flowers and leaves since those contain the highest concentration of cannabinoids, especially ∆^9^-THC and CBD (Jin et al. 2020; Kleinhenz et al. 2020a). The NDF and ADF content in SHB might vary depending on the maturity stage of the plant and the biomass used for the cannabinoid extraction (whole plant vs. flower and leaves). The NDF content of SHB from three sources in Oregon varied between 23.4% and 43.7% (Fig. 3). SHB is rich in Ca, P, Zn, Cu, and Mn, which are 2- to fivefold higher than alfalfa (Table 1). Some trace minerals are essential for dairy cows, especially their roles in the immune system and hoof health (Faulkner et al. 2017; Zhao et al. 2015). However, a high level of Ca in SHB could be a concern when fed to dry dairy cows, especially Jersey cows, due to the importance of a low Ca in the diet during the prepartum to minimize the risk of milk fever (Oetzel and Madison 2011). However, the level of K in the diet appears to be more important than the level of Ca in inducing metabolic alkalosis that can disrupt the Ca homeostasis, and the calculation of the dietary cation–anion difference in the diet contains K in the numerator and not Ca (Goff and Horst 1997). High Fe content can also be deleterious as it can induce toxicity when given at higher amounts than recommended levels for ruminants (NASEM 2021). Note that potentially hazardous materials or substances such as acetone, terpenes, heavy metals, and pesticides might be left in the SHB as a result of the extraction process. Our data indicated that those hazardous substances were not detected (< limit of detection) and below the maximum tolerable limit for heavy metals such as arsenic, cadmium, lead, and mercury from the SHB obtained from processors in Oregon, USA (Parker et al. 2022). However, it is advisable to analyze such substances from other processors to ensure the safe use of SHB.

#### Amino acid profile

Table 2 summarizes the concentration of amino acids in dehulled hempseed, hemp hurds, HSB, and SHB, as well as SBM and alfalfa, which are used as comparators.

**Table 2:** Amino acids profile of hemp products compared with soybean meal and alfalfa

Amino acids, % DM	Hemp byproducts				Comparators		
	HH	HS	HSB	SHB	SBM	DDGS	Alfalfa
*Essential AA (EAA)*							
Arg	0.94	2.79	3.91	0.97	3.43	1.46	0.85
His	0.25	0.73	0.93	0.37	1.27	0.92	0.32
Ile	0.39	0.96	1.45	0.68	2.16	1.24	0.82
Leu	0.71	1.62	2.35	1.18	3.57	3.62	1.46
Lys	0.33	0.81	1.32	0.96	2.92	1.07	0.93
Met	0.18	0.59	0.88	0.29	0.67	0.63	0.25
Tyr	0.40	1.37	1.15	0.58	1.66	-	0.63
Val	0.60	1.27	1.91	0.88	2.26	1.63	1.21
Total EAA	3.80	10.1	13.9	5.91	17.9	13.13	6.47
*Non-essential AA (NEAA)*							
Ala	0.40	1.00	1.61	0.87	2.05	2.13	1.02
Asp	0.90	2.42	3.66	2.76	5.27	1.96	2.03
Cys	0.18	0.21	0.70	0.27	0.72	0.59	0.25
Glu	1.19	4.07	6.03	2.00	8.34	4.02	1.88
Gly	0.41	1.20	1.66	0.93	1.99	1.27	0.89
Phe	0.53	1.26	1.62	0.92	2.37	-	0.94
Pro	0.69	1.03	1.59	0.90	2.35	2.66	0.83
Ser	0.42	1.15	1.73	0.80	2.31	1.25	0.83
Thr	0.36	0.77	1.35	0.68	1.83	-	0.78
Trp	0.06	0.15	0.39	0.33	0.63	-	0.29
Total NEAA	5.14	13.3	20.3	10.5	27.9	13.88	9.74
* Branch-chain*	1.70	3.85	5.71	2.74	7.99	6.49	3.49
* Lys/Met*	1.83	1.37	1.50	3.31	4.36	1.71	3.72
* Total AA*	8.94	23.40	34.20	16.41	45.8	27.71	16.21

Data are derived from: (House et al., 2010; Mjoun et al., 2010; Parker et al., 2022; Mohamed et al., 2023; Winders et al. 2023)

*DDGS* Distillers dried grains with solubles, *HH* hemp hurd, *HS* hemp seed, *HSB* hemp seed byproducts, *SHB* spent hemp biomass, *SBM* soybean meal

##### Hempseed byproducts

The amino acid (AA) concentration of HSB is higher than HS after dehulling due to the elimination of fiber content. As the content of CP is lower, the amount of AA of HSB is lower than SBM (34.2 vs. 45.8%). However, compared to other grains or byproducts like corn and distiller’s dried grains with soluble (DDGS), HSB contains higher AA (Winders et al. 2023). It is comparable to canola and rapeseed meals (Berrocoso et al. 2015; Fontaine et al. 2001; Maxin et al. 2013). Compared to DDGS as one of the most common grains used in ruminants’ ration in North America, HSB contains a higher proportion of Arginine (Arg), lysine (Lys), methionine (Met), aspartate (Asp), glutamic acid (Glu), glycine (Gly), phenylalanine (Phe), and serine (Ser). Still, the proportion of total branch chain AA (BCAA) was lower. Leucine (Leu), alanine (Ala), and proline (Pro) are the only AA in HSB with a lower proportion compared to DDGS. The overall higher AA content of HSB compared to DDGS resulted in higher plasma Arg, Lys, Met, and Glu in heifers (Winders et al. 2022b), indicating the higher AA uptake from dietary HSB. The higher plasma Lys and Met is of interest as these AA are known to be the most limiting AA, and a higher supply of Lys and Met could be beneficial for ruminants to meet their Lys and Met requirement. (Giallongo et al. 2016; Räisänen et al. 2021). The lower BCAA of HSB compared to DDGS and SBM might be undesirable and might have some impact on ruminant production because BCAA is also considered as important as Lys and Met and plays an important role in various cellular processes and milk protein synthesis (Appuhamy et al. 2011). Overall, despite the relatively lower BCAA content of HSB compared to DDGS and SBM, HSB could serve as a valuable alternative amino acid source in concentrates for major ruminant animals.

##### Spent hemp biomass

In terms of amino acid composition, SHB is largely comparable to alfalfa, with some differences. Alfalfa tends to have higher proportions of His and Lys compared to SHB. Conversely, SHB contains higher proportions of Cys and Glu compared to alfalfa. Lys is considered, together with Met, among the most essential AA for milk synthesis (Robinson et al. 2010), although both the concentration of the two AA and their ratio are important to maximize milk protein synthesis (Awawdeh 2016). A Lys-to-Met ratio of 3:1 has been shown to optimize milk protein synthesis in dairy cows (Awawdeh 2016). Interestingly, the SHB has a Lys/Met ratio of almost exactly 3:1. Alfalfa and SHB have a similar abundance of essential AA and BCAA. Those AA are important for their role in activating the mTOR pathway, the master regulator of milk protein synthesis, but also for the synthesis of lactose and fat (Osorio et al. 2016). This holds particularly true for the branched-chain amino acid Leu, which is either equal to or slightly higher in proportion in SHB compared to alfalfa. In beef cattle or meat-producing ruminants, corn silage or alfalfa hay are among the most common forages in diets. While alfalfa hay may have a relatively similar AA profile to SHB, as discussed above, corn silage typically has a poor AA profile. Mariz et al. (2018) reported the Lys, Met, and BCAA of corn silage were 0.27, 0.05, and 0.99, respectively, which were overall poorer than SHB. The Lys/Met ratio of corn silage in that study was 5.4, which is higher than 3 and is unbalanced for ruminants. Despite the lack of studies evaluating the impact of SHB on beef cattle, the AA profile of SHB is more desirable than corn silage but comparable to alfalfa hay. Nevertheless, it should be noted that different extraction techniques may give different AA content. To our knowledge, there is no study reporting the effects of extraction methods including the solvent and temperature interactions on the AA content of SHB.

#### Fatty acid profile

Among fatty acids (FA) found in the hemp plant, α-linolenic acid stands out as the most abundant, comprising approximately 50% of the total FA. Following closely behind is linoleic acid, making up between 10 and 20% of the total FA content in the whole plant (Peiretti 2009). Exploration of the nutritional profile of hemp seed-related products from various regions indicate an overall high content of polyunsaturated fatty acids (PUFA) (Cattaneo et al. 2021; Farinon et al. 2020; Gibb et al. 2005; Jing et al. 2017; Karlsson et al. 2010; Mierliță 2018; Vonapartis et al. 2015). Approximately 30% of hemp seed components are lipid fractions, just below some major oil seeds such as sunflower and flaxseed, where the fat content is > 35% (Callaway 2004).

**Fig. 4:**
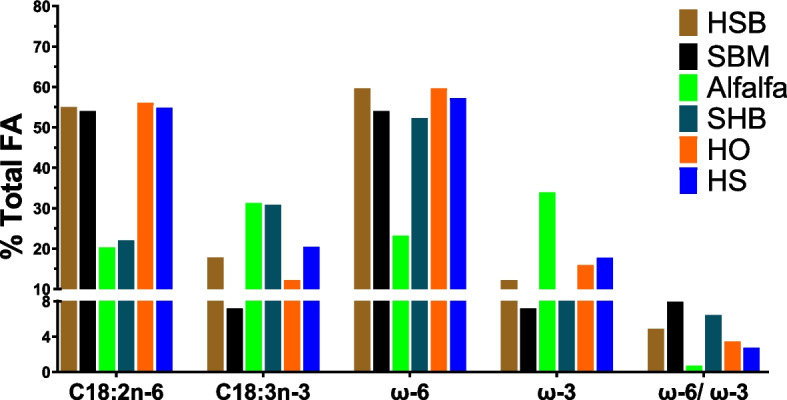
Fatty acids profile of various hemp products compared with soybean meal and alfalfa (Note: HSB = hemp seed byproduct, SBM = soybean meal; SHB = spent hemp biomass, HO = hemp oil, HS = hemp seed). Data are derived from: (Turner et al., 2008; Pojić et al., 2014; Irawan et al., 2023; Mohamed et al., 2023)

The FA profiling of various hemp byproducts, SBM, and alfalfa is presented in Fig. 4. Similar to hemp oil and hempseed, the SHB is rich in unsaturated FA with proportionally higher C18:3 n-3 (approximately 30%) compared with HSB (< 20%) and SBM (< 10%) but comparable with alfalfa. However, the fat content of SHB is considerably higher than that of alfalfa (Table 1). This is not surprising since the SHB and alfalfa are mainly leaves, and the thylakoid membranes where photosynthesis happens are rich in ω3 (Buccioni et al. 2012). The ratio ω6/ω3 in SHB is around 0.60, which is highly desirable and known to be important in preventing diseases in humans (Simopoulos 2010) since ω3 FA are used in cells as precursors for the synthesis of anti-inflammatory compounds while ω6 pro-inflammatory eicosanoid lipid mediators (Calder 2013). Although data on ω6/ω3 of HSB is lacking, SHB has a desirable FA profile due to the high content of C18:3 n-3 (> 10%) and total PUFA (> 70%), higher than SBM (approximately 60% PUFA).

PUFA possesses many nutritional benefits related to the improvement of metabolism, health, and product quality, as well as for maintaining pregnancy and reproduction, as comprehensively reviewed by Moallem (2018). Increased levels of linoleic acid and α-linolenic acid are key nutritional components in hempseed and its byproducts, offering significant benefits for both poultry (Attia et al. 2022; Jing et al. 2017; Semwogerere et al. 2020) and ruminants (Abrahamsen et al. 2021b; Nchama et al. 2022; Šalavardić et al 2021; Zhou et al. 2022). Despite the data available so far indicating a desirable profile of FA of the various hemp byproducts as feed ingredients for animals, this profile can be affected by the extraction method used and the variety of hemp.

### Bioactive compounds and anti-nutritional factors

Hemp is a source of highly diverse phytochemicals with more than 500 identified compounds consisting of approximately 125 cannabinoids, 120 terpenes, 42 phenolics, 34 flavonoids, 2 alkaloids, and many other compounds (Al Ubeed et al. 2022).

#### Antinutritional factors

**Fig. 5:**
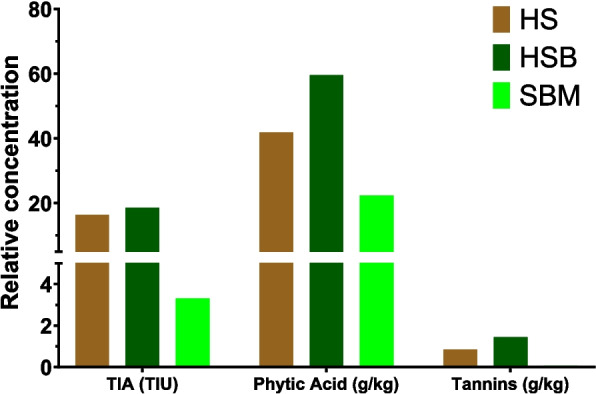
Antinutritional factors of hempseed and hempseed meal compared with soybean meal (Note = HS = hemp seed, HSB = hemp seed byproduct, SBM = soybean meal, TIA = trypsin inhibitor activity). Data are derived from (Pojić et al., 2014; Hong et al., 2015; Galasso et al., 2016; Farinon et al., 2020; Alonso-Esteban et al., 2022; Song et al., 2022)

The hemp plant contains a large number of bioactive compounds (Cerino et al. 2021). Hemp seed contains several antinutritional compounds such as protease inhibitors and phytic acid (Alonso-Esteban et al. 2022b). The latter is in higher amounts compared to soybean meal (Fig. 5). The high levels of protease inhibitors can negatively affect the growth performance in monogastric animals, such as poultry. However, this should not affect ruminants because phytic acid is degraded by microbial phytase in the rumen and can contribute to phosphorus supply (Spears 2003). Furthermore, ruminants tolerate trypsin inhibitors found in many grains, including HSB. The activity of trypsin inhibitors is deactivated by rumen microbes within 24 h of incubation (Hoffmann et al. 2003). There is currently a lack of literature reporting on antinutritional compounds in SHB. However, our recent study revealed the absence of pesticides and mycotoxins in SHB, along with very low levels of heavy metals (Parker et al. 2022).

#### Cannabinoids

Chemically, cannabinoids are part of the family of C21 terpenophenolic compounds that are synthesized in glandular trichomes of hemp and, thus, present in the female inflorescence and leaves (Jin et al. 2020; Ning et al. 2022; Pojić et al. 2014) but not in the seed; thus, detectable cannabinoids in hempseed and its byproducts are the results of contamination during the plant processing (Jang et al. 2020)***.***

There are ten major subclasses of cannabinoids namely cannabichromene (CBC), CBD, cannabielsoin (CBE), cannabigerol (CBG), cannabicyclol (CBL), cannabinol (CBN), cannabinodiol (CBND), cannabitriol (CBT), ∆^8^-THC, and ∆^9^-THC (Radwan et al. 2021). The various cannabinoids are all derived from their parent compound, cannabigerolic acid (CBGA) (Al Ubeed et al. 2022). Among cannabinoids, CBD and THC are the most important and widely investigated. In contrast, the former is not psychoactive, and the latter is a psychoactive compound with known adverse effects on the neural system. Commercial products based on these compounds are available in the market. The two cannabinoids are approved as 50% mixed in Europe to treat multiple sclerosis (Sativex Oromucosal Spray; nabiximols®) and as 98% pure CBD to treat epilepsy in the USA (Epidyolex®) (Cerino et al. 2021).

Industrial hemp typically contains high concentrations of CBD but low concentration of THC (Arango et al. 2024; Wang et al. 2023). The concentrations are highly variable for CBD (< 0.01–1.0% w/w) and CBDA (< 0.5–16% w/w for CBDA) depending on cultivars, flowering phases, part of the plant, and geographical and agroclimatic conditions (Glivar et al. 2020). On the other hand, THC concentrations must be below 0.3% w/w in regulations of many countries (Glivar et al. 2020; Kleinhenz et al. 2020b; Wagner et al. 2022). After extraction, trace amounts of various compounds can remain in the biomass, even with highly optimized extraction methods (Qamar et al. 2021; Al Ubeed et al. 2022). For instance, > 5 mg/g (0.5%) CBD remains detectable (but not THC) in post-extracted hemp dust (Attard et al., 2018), and 0.2% CBD and > 2% CBDA also remain in hemp threshing residues (Kitrytė et al. 2018).

Cannabinoid content on various hemp byproducts is reported in Table 3. Cannabinoids are synthesized in glandular trichomes, an aerial part of the plant, and are stored in the seedless female flowers (Jin et al. 2020). Cannabinoids are also found in the leaves during vegetative stages and gradually decrease during the flowering stages (Aizpurua-Olaizola et al. 2016). This is why the highest content of cannabinoids is found in flowers, followed by the leaves. Different from the Cannabis-drug type, which can contain nearly 20% total cannabinoids in the inflorescence and < 5% in the leaves (Jin et al. 2020), hemp contains approximately between 5–6% total cannabinoids in the flower (Hillig and Mahlberg 2004) with less cannabinoids in the leaves (Fetterman et al. 1971). In another study, Kleinhenz (2020a) reported relatively higher cannabinoids in the leaves (5.2%) than in flowers (4.3%) of industrial hemp. The higher content of the leaves was because the leaves were dried while the flowers were unprocessed; it likely was higher in the flowers when expressed as a dry weight basis. After extraction, SHB still contains a relatively high level of cannabinoids, as observed in the samples used in our experiments (Fig. 3), with levels of total cannabinoids ranging from 1.2% to 3.0%, with the highest detectable level of ∆^9^-THC being 0.03%. In China, a lower concentration of CBD (0.03%) in SHB was reported with no detectable ∆^9^-THC (Wang et al. 2023).

**Table 3:** Cannabinoid profiles of hemp, including leaves and flowers, and hemp byproducts

Cannabinoids^1^ mg/g	Hemp products^2^					
	Flower	Leaves	Plant	SHB	HSB	HS
CBC	0.51	0.42	0.19	0.55	-	-
CBCA	0.03	4.04	0.85	1.55	-	-
CBD	4.13	3.35	0.40	5.20	0.0004	0.002
CBDA	32.9	36.9	4.87	8.67	-	0.034
CBG	0.23	0.29	0.03	-	-	-
CBGA	1.94	1.79	0.26	-	-	-
CBN	0.03	0.004	0.01	-	-	-
Δ^9^-THC	0.66	0.57	0.10	0.30	-	-
THCA	3.38	4.61	0.31	0.40	-	0.002
THC Total	4.05	5.18	0.41	0.70		0.12
Total	43.2	52.0	7.03	16.7	0.0004	0.15

^1^*CBC* cannabichromene, *CBCA* cannabichromene acid, *CBD* cannabidiol, *CBDA* cannabidiolic acid, *CBG* cannabigerol, *CBGA* cannabigerolic acid, *CBN *cannabinol, ∆^9^-THC = ∆^9^-tetrahydrocannabinol; THCA = tetrahydrocannabinolic acid

^2^*SHB* spent hemp biomass, *HSB* hemp seed byproducts (hemp seed cake or meal), *HS*, hemp seed, the leaves were dried samples while the flowers were unprocessed samples

Data were derived from: (Galasso et al. 2016; Kleinhenz et al. 2020a; Mohamed et al. 2023; Parker et al. 2022; Peiretti 2009; Song et al. 2022; Wagner et al. 2022; Wang et al. 2023)

#### Other phytochemicals: essential oil, flavonoids, and terpenes

Hemp is rich in bioactive compounds besides cannabinoids, such as essential oil, flavonoids, condensed tannins, glycosides, saponins, and terpenes (Isidore et al. 2021). Many of those can benefit animals and humans (Cerino et al. 2021). These compounds can be of potential interest due to their beneficial effects on enhancing rumen fermentation and ruminant production (Health et al. 2015; Olagaray and Bradford 2019; Yanza et al. 2021). However, few studies have investigated the roles of bioactive compounds in hemp byproducts on livestock. Among the byproducts, HSB may be promising to enhance the health value of meat by increasing the relative composition of desirable flavor volatile compounds and meat protein self-life stability (Semwogerere et al. 2023a). In these studies, HSB contained 0.25 g/kg total tannins and 0.36 g/kg total phenolic compounds, two times higher than soybean meal. Phenolic compounds are naturally present in hemp seed with antioxidants and anti-inflammatory properties (Chen et al. 2012; Rea Martinez et al. 2020). Studies investigating bioactive compounds extracted from hemp seed and hemp seed meal suggested the potential inhibition of inflammatory cytokines (IL-6 and TNF-α) in LPS-activated human monocytes (Rea Martinez et al., 2020) and mouse LPS-activated macrophages (Lin et al. 2021). Another study reported that hemp seed contains relatively similar values of 0.4 g/kg total phenols, 0.3 g/kg tannins, and various classes of tocopherols ranging from 0.6 to 15.3 µg/g (Semwogerere et al., 2023b). In a recent study, feeding hemp hay to grazing goats also resulted in increased antioxidant potential and a decrease in serum level of reactive oxygen metabolite-derived compounds and TNF-α, suggesting that hemp hay might exert antioxidant and anti-inflammatory effects (Zicarelli et al. 2025).

Tocopherols, as the essential vitamin E, when provided above dietary requirements can have health benefit, including reducing inflammation and cholesterolemia but can also act as an anti-fungal and anti-viral agent (Vecka et al. 2019), and it is known to benefit ruminants (McDowell et al. 1996). The antioxidant potential of HSB has also been reported. For instance, the 2,2-Diphenyl-1-picrylhydrazyl (DPPH) radical-scavenging activities, one major method to indicate antioxidant activity, is considerably high in hempseed ranging from 69 to 92% (Ning et al. 2022). Moreover, CBD, CBDA, and ∆9-THC also have antioxidant activity (Dawidowicz et al. 2021). Hempseed contains several polyphenols including caffeoyltyramine, and cannabisin A, cannabisin B, and cannabisin C and these total polyphenols were reported to have high correlation with DPPH scavenging activity (*r* > 0.90) (Frassinetti et al. 2018). Cannabisin B has a significant DPPH scavenging activity (Chen et al. 2012).

### Impact of hemp byproducts on ruminant production: a meta-analysis

A meta-analytical approach was carried out to systematically summarize findings related to hemp byproducts on ruminants instead of relying on a narrative review. This approach also facilitates minimizing authors’ bias in interpreting data from multiple studies. We gathered 26 available studies so far for the meta-analysis (Table 4) with detailed methodology is provided in the supplementary file 1. For response parameters that are very limited in the literature, a narrative review was employed.

**Table 4:** Studies evaluating hemp byproducts on ruminant animals

Study	Country	Byproducts	Animal	Inclusion, % DM	Evaluated parameters	Reference
1	Sweden	HSB	Dairy bull & calves	12.6–20	Performance, nutrient intake, carcass	Hessle et al. (2008)
2	Sweden	HSB	Beef steers	12.6	FA profile	Turner et al. 2008)
3	Sweden	HSB	Dairy cows	14.3–31.8	Milk production and composition	Karlsson et. (2010)
4	Sweden	HSB	Lambs	21.8	Production performance	Karlsson et al. (2011)
5	Sweden	HSB	Lambs	21.8	FA profile	Turner et al. (2012)
6	Croatia	HSB	Lambs	12	Performance, blood profile, antioxidant status	Antunović et al. (2020)
7	Croatia	HSB	Dairy goats	0.6–1.2	Milk production and FA profile	Šalavardić et al. (2021)
8	USA	HSB	Goats	10–30	Production performance, blood biochemistry, rumen fermentation	Abrahamsen et al. (2021b)
9	South Africa	HSB	Goats	2.5–10	Production performance, carcass, and meat quality	Semwogerere et al. (2023b)
10	Italy	HSB	Dairy cows	4.5	Production performance and meat quality	Nchama et al. (2022)
11	USA	HSB	Finishing heifers	20	Production performance, plasma amino acids profile	Winders et al. (2022b)
12	USA	HSB	Goats	10–30	Carcass and meat quality	Gurung et al. 2022)
13	USA	SHB	Lambs	10–20	Production performance, meat and carcass, blood biochemistry	Parker et al. (2022)
14	Germany	HS	Dairy cows	8.8	Milk production and composition, activity, cannabinoid transfer	Wagner et al. (2022)
15	USA	HSB	Beef steers	20	Rumen fermentation, nutrient utilization	Winders et al. (2023)
16	South Africa	HSB	Goats	2.5–10	Meat antioxidant quality and volatile compounds of the meat	Semwogerere et al. (2023a)
17	USA	SHB	Dairy cows	13	Milk production and quality, FA profile, nitrogen use, blood biochemistry, methane emission	Irawan et al. (2024)
18	China	SHB	Dairy cows	6–10.8	Milk production, rumen fermentation, immunity	Wang et al. (2023)
19	Canada	HSB	Dairy cows	6.25–10.2	Rumen fermentation, digestibility, and cannabinoid excretion	Addo et al. (2023a)
20	Canada	HSB	Dairy cows	7.5–15	Production performance, FA profile, cannabinoids excretion	Addo et al. (2023b)
21	Greece	HSB	Dairy cows	3.5	Milk production, milk quality, health parameters	Kalaitsidis et al. (2023)
22	Romania	HS	Goats	250 g/d	Milk production, milk fatty acid, antioxidant	Mierlita et al. (2023)
23	Italy	HP	Goats	250 g/d	Milk production & fatty acid	Iommelli et al. (2024)
24	USA	HSB	Bulls	10	Production and health parameters	McGovern et al. (2024)
25	Italy	HP	Goats	250 g/d	Milk production & fatty acid	Amato et al. (2024)
26	China	HP	Goats	5–20	Production, rumen fermentation	Ran et al. (2024)

*HP* Hemp plant material containing cannabinoid including hemp leaves hemp inflorescence hemp hay hemp silage, *HSB* hempseed byproduct (hempseed meal or hempseed cake), *SHB* spent hemp biomass, an extracted byproduct from the hemp plant

#### Dry matter intake and production performance

**Fig. 6:**
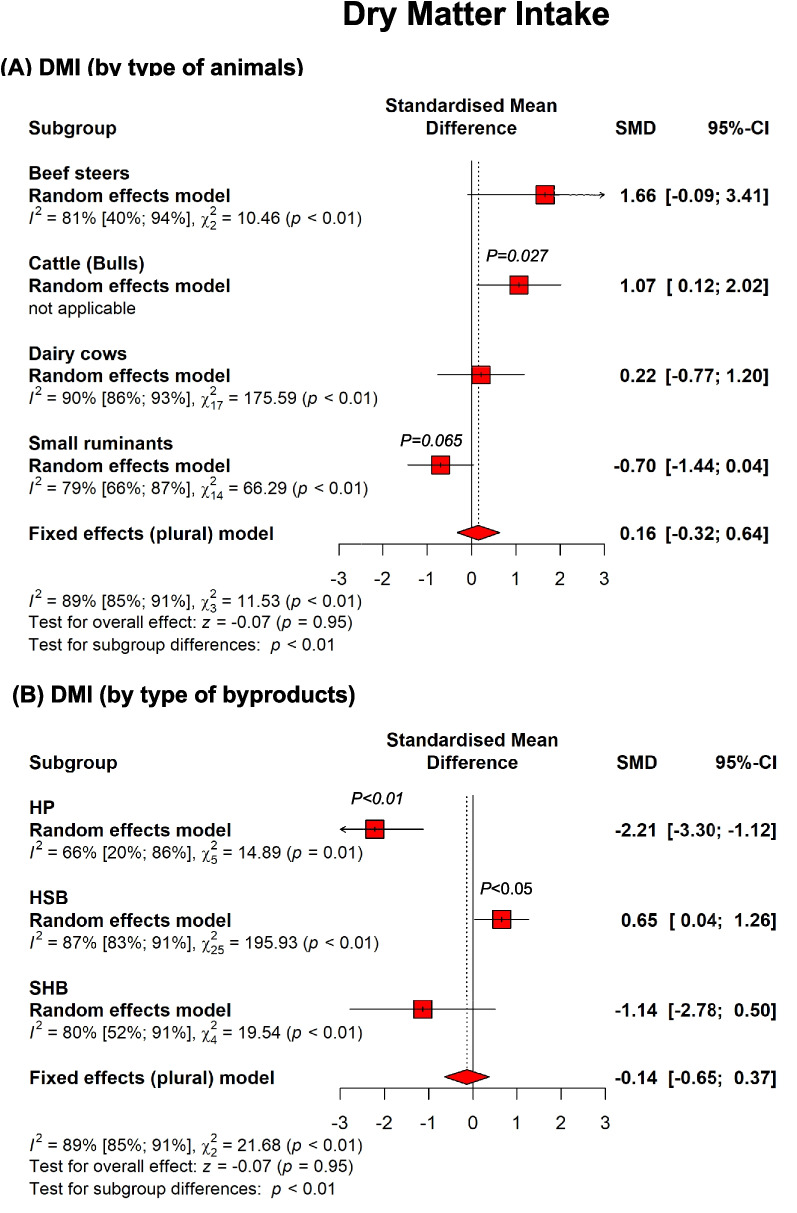
Summary of subgroup meta-analysis of the effects of hemp plant (HS), hempseed byproducts (HSB), and spent hemp biomass (SHB) on dry matter intake in ruminants

Overall, the inclusion of hemp byproducts tended to increase the DMI in beef cattle (*P* = 0.06) but tended to decrease DMI in small ruminants (*P* = 0.065) without affecting DMI in dairy cows (Fig. 6). The increase in DMI in beef cattle was mainly associated with HSB (*P* = 0.052). Regarding SHB, there are two studies in lactating dairy cows and one study in lambs indicating that SHB has low palatability, possibly due to the distinct “skunky” odor of cannabis, as previously suggested (Irawan et al. 2024). Feeding 20%, SHB reduced DMI by 20% in finishing lambs during the first month of feeding, while no effect on DMI was found during the second month of feeding SHB (Parker et al. 2022). In dairy cows, adding SHB to the diet reduced DMI by 8–9% when included at 7.5% DM (Irawan et al. 2024) or 13% DM in the diet. Despite the reduced DMI, weight gain and milk yield were unaffected. Another recent study reported a similar detrimental effect of hemp on DMI, where the inclusion of 8% hemp silage on lactating dairy cows decreased not only DMI but also milk yield (Wagner et al. 2022). The authors inferred the effect of the levels of cannabinoids in the hemp silage; however, this does not appear to be entirely consistent with prior data, as previously argued (Irawan et al. 2024). Dry matter digestibility was not affected by hemp byproducts, but NDF digestibility was decreased (*P* = 0.04) (Supplementary Table> 1).

**Fig. 7:**
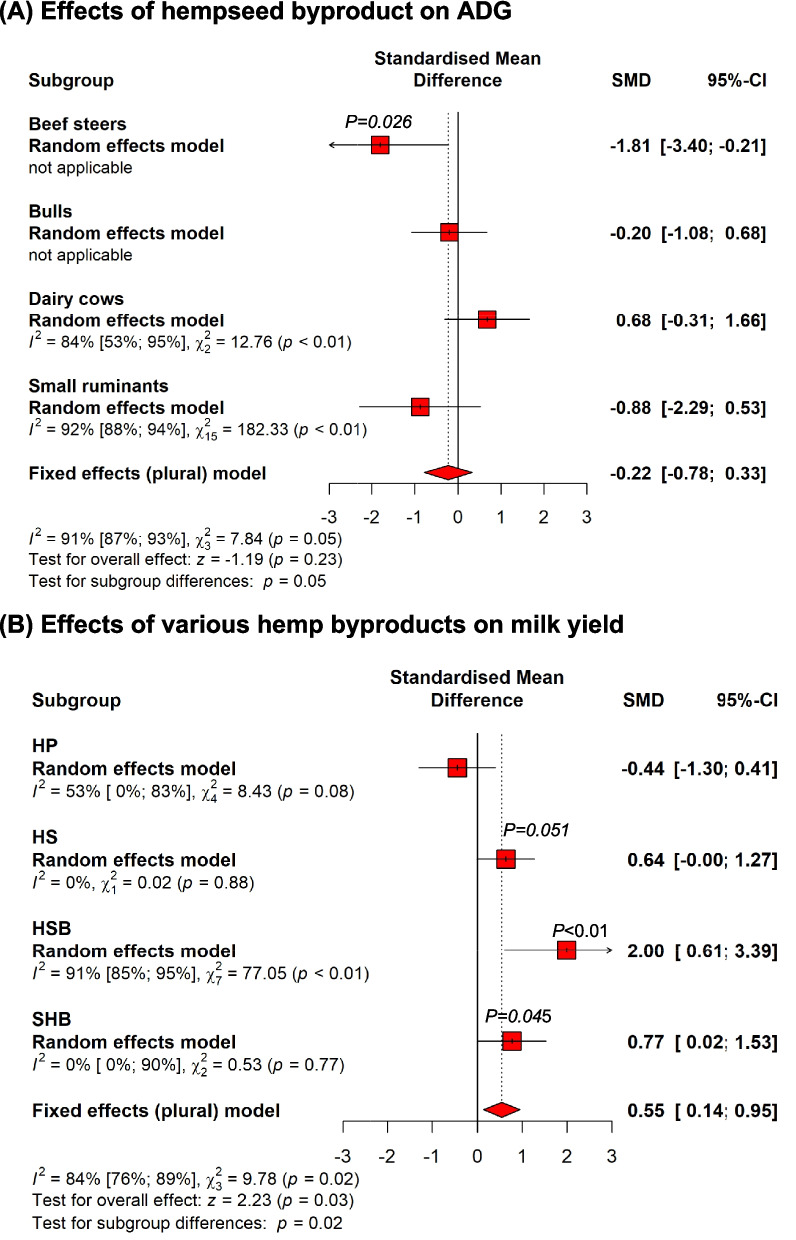
Summary of subgroup meta-analysis of the effect of hempseed byproduct on average daily gain of ruminants (**A**); and effects of various hemp byproducts including hemp plant (HP), hemp seed byproducts (HSB), and spent hemp biomass (SHB) on milk yield of dairy ruminants (**B**)

Overall, feeding HSB did not affect the average daily gain (ADG) of dairy cows and small ruminants but decreased ADG of beef cattle (*P* = 0.03) (Fig. 7A). In a study using goats, dietary HSB between 10 and 30% in isocaloric and isonitrogenous diets was reported to have a linear decrease of ADG in goats from 11 to 27%, likely due to the high level of lignin (12.8%) in the HSB (Abrahamsen et al., 2021). The high level of fiber in non-dehulled hemp seed byproducts typically used (Karlsson et al. 2010; Šalavardić et al., 2021; Winders et al. 2022b) is likely the cause of the negative effect of HSB on ADG.

Overall, hemp byproducts tend (*P* = 0.06) to increase milk yield, especially in small ruminants (Fig. 7B). In dairy ruminants, the inclusion of hemp plant material reduced milk yield (SMD = −1.22; *P* = 0.02) while dairy cows fed SHB and HSB showed higher milk yield (*P* < 0.05; Fig. 7B). Regarding the SHB, limited studies included in the meta-analysis is a major limitation whereas the finding was contrary in one study to another (Irawan et al. 2024; Wang et al. 2023). In dairy cows, a quadratic effect on milk yield was observed with HSB whereas 14% dietary HSB increased the milk yield by 14% but higher doses of HSB in the diet did not affect milk yield (Karlsson et al., 2010). Note should be taken, however, that between-studies heterogeneity was high (> 75%). In the studies reporting production performance, discrepancies are identified regarding the effects of HSB where several experiments reported increased milk yield in dairy goats (Šalavardić et al., 2021) and weight gain in lambs (Antunović et al. 2020) while other studies reported decreased weight gain (Turner et al. 2008; Winders et al. 2022b). Overall, the effects of hemp byproducts depended on the types of byproducts and types of ruminants whereas HSB appeared to have more palatable and desirable effect on ruminants’ performance than SHB.

#### Carcass, meat, and milk quality

In meat-producing ruminants, incorporating various hemp byproducts does not impact carcass weight and dressing percentage (Supplementary Table 1) but has some favorable effects on carcass quality, especially on the FA profile of the meat. Few studies reported that HSB inclusion does not affect the content of moisture, fat, and protein in the meat (Gurung et al. 2022; Nchama et al. 2022; Semwogerere et al. 2022).

##### Meat animals

**Fig. 8:**
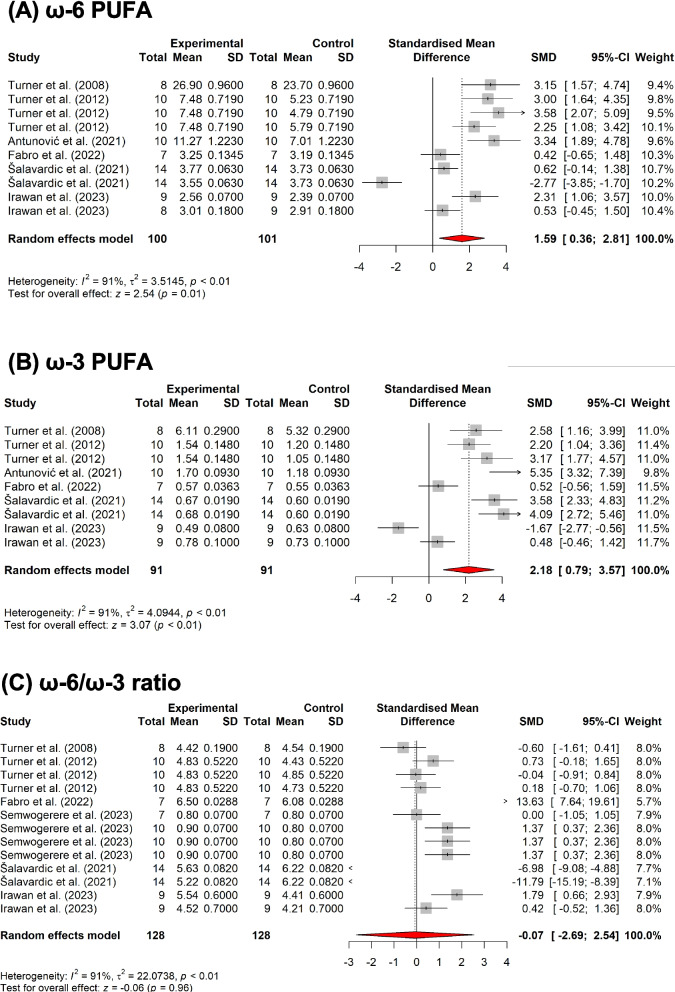
Results of subgroup meta-analysis of the effects of hemp silage (HS), hemp seed byproducts (HSB), and spent hemp biomass (SHB) on omega-3 and omega-6 fatty acids in ruminants

As discussed earlier, hemp byproducts have favorable FA profiles, and various compounds serve as antioxidant properties that are advantageous to improve meat quality. It was empirically evident that feeding HSB led to higher proportions of omega-3 fatty acids in the meat of ruminants. As shown in Fig. 8A and Fig. 8B, feeding HSB to cattle increased n-6 FA and n-3 PUFA proportions in the meat. However, the n-6/n-3 ratio was not affected (Fig. 8C). Finishing steers that were fed 12% HSB had a larger proportion of C18:3 n-3, C18:3 n-6, and C18:2 n-6 in *M. longissimus dorsi* but did not affect the n-6/n-3 ratio compared to feeding soybean meal (Turner et al. 2008). In a similar study, feeding 12% HSB led to the enrichment of docosahexaenoic acid (DHA) and overall n-3 contents of *musculus semimembranosus* compared to the same proportion in the diet of soybean meal in lambs (Antunović et al. 2020). Studies in small ruminants feeding HSB resulted in increased omega-3 in the meat from 24 to 44% (Antunović et al. 2020; Semwogerere et al. 2023b; Turner et al. 2008).

A linear increase in the levels of n-3 PUFA, as well as n-6 PUFA, was observed in goat meat when fed with HSB (Semwogerere et al. 2023b). The increase in these PUFA levels is beneficial since n-3 fatty acids can reduce the risk of cardiovascular diseases (Khan et al. 2021; Mozaffarian and Wu 2011), while n-6 PUFA can help with reducing depression and improving fertility (Vahmani et al. 2020). A high proportion of HSB in the diet of lambs significantly increased by 21.8% the concentration of 18:3 n-3 and DHA in the *M. longissimus dorsi* (Turner et al. 2012).

There is only one study documenting the effect of feeding SHB on carcass quality and meat (Parker et al. 2022). In that study, feeding up to 20% SHB in the diet of finishing lambs did not affect the FA profile of muscle or adipose tissue. The only noticeable effects on carcass and meat quality were an increase in purge loss and meat cook loss when lambs were fed a diet containing 20% SHB compared to the control group.

##### Dairy animals

Milk fat and milk protein percentages were decreased (*P* < 0.05) by feeding hemp byproducts, while lactose was unaffected (Supplementary Table 1). Few studies have documented the effects of HSB and SHB on milk quality and yield in dairy ruminants. Feeding 12% HSB to dairy goats to replace soybean meal reduced the proportion of C18:0 and the linoleic/linolenic acid ratio and tended to increase the n-3 proportion in milk but decreased % lactose and increased milk urea (Šalavardić et al. 2021). Wang et al. (2023) reported that feeding 11% SHB derived from ethanol extraction of industrial hemp did not affect the percentage and yield of milk fat, protein, and lactose in lactating Holstein dairy cows. In our recent study, lactating cows that consumed 7.4% SHB in their diets tended to produce milk with a lower percentage of milk fat, while protein and lactose levels remained unaffected (Irawan et al. 2024). Although the proportion of FA in milk was not affected by feeding SHB, an improved calculated hypocholesterolemic/hypercholesterolemic (h/H) index was detected (Irawan et al. 2024). Cumulatively, the majority of findings indicated no negative effect of incorporating hemp byproducts into the diets of ruminants while some positive alterations of FA profile were observed, underscoring their beneficial health effect.

#### Antioxidant status

Hemp byproducts are a source of various phytochemicals with antioxidant properties, including phenolic compounds, terpenes, and cannabinoids (Cantele et al. 2020; Isidore et al. 2021; Serventi et al. 2023; Vitorović et al. 2021; Zhang et al. 2022). A recent study revealed the presence of 201 flavonoids, 86 alkaloids, and 149 phenolic acids with high antioxidant potentials in hemp seed (Ning et al. 2022). Among those, ferulic acid-hexoside and syringic acid have high antioxidant capacity (Alonso-Esteban et al. 2022a). The same study reported the potential of dehulled hemp to inhibit lipid peroxidation and malondialdehyde formation, corroborating results from a previous study (Hong et al. 2015). In addition to the antioxidant potential of polyphenols, cannabinoids also possess antioxidant potential. CBG, CBD, Δ^9^-THC, CBN, CBGA, CBDA, and Δ^9^-THCA have a comparable antioxidant capability with vitamin E and higher scavenging free radicals’ activity than Trolox, a water-soluble analog of vitamin E, as well as effective inhibition of oxidative processes (Dawidowicz et al. 2021). Supplementation of a relatively low amount of CBD improved the antioxidant status of murine microglial cells, likely due to the scavenging of free radicals as well as the activation of the transcription factor NRF2 (Juknat et al. 2013).

The FA compositions of hemp can also enhance the flavor and antioxidant capacity of meat, as evidenced by animals fed with hemp seed-based diets (Semwogerere et al. 2023b). An increase in HSC supplementation in goats had a linear improvement of the antioxidative potential in blood, liver, and meat (Semwogerere et al. 2023a). Results on the effect of HSB on the antioxidant status of the animals in literature are not entirely consistent. For instance, Šalavardić et al. (2021) conducted a feeding study using HSB with a relatively similar nutritional profile, especially ether extract and n-3 proportion with a prior study (Semwogerere et al., 2023b) but the results regarding the antioxidant status of the animals was inconsistent. In the former study, goats fed 12% HSB had a lower GPx activity compared to the control group, while feeding 6% HSB did not affect GPx activity. Feeding 10 or 20% SHB in lambs improved the antioxidant status of the animals (Parker et al. 2022). In lactating dairy cows, feeding SHB did not affect the antioxidant status of the animals (Irawan et al. 2024; Wang et al. 2023), but in our study, we detected a higher concentration of reactive oxygen metabolites (ROM) in plasma (Irawan et al. 2024). The variations in the results could be attributed to differences in species or animal products (such as meat versus milk).

#### Health parameters

Studies evaluating the health parameters in ruminants fed hemp byproducts are extremely limited. HSB inclusion in ruminants’ diets appeared to be safe, as no health issues have been reported so far. Experiments evaluating HSB on the feeding activity of heifers did not observe any difference compared to DDGS (Winders et al. 2022a).

Wang et al. (2023) and our laboratory (Irawan et al. 2024) have also thoroughly evaluated a plethora of health parameters measurement in blood, including complete blood count and parameters related to immunity, liver health, and inflammation, and concluded that almost all those parameters were unaffected by feeding SHB to lactating dairy cows. We (Irawan et al. 2024) reported, however, some effects on the drug clearance ability of the liver. The latter was also consistent with our study of lambs (Parker et al. 2022). In both studies, bilirubin concentration in plasma was increased in animals fed SHB without any indication of liver issues, according to other liver-related parameters measured, suggesting a reduced ability for liver clearance. This consistent phenomenon was suggested to be associated with the inhibition by cannabinoids, specifically CBD, of UDP-glucuronosyltransferase 1 A1, the enzyme that plays a major role in drug clearance and bilirubin metabolism in the liver (Parker et al. 2022); however, a reduced clearance of drugs by the liver of animals fed SHB need to be demonstrated.

Feeding HSC up to 12% had no effect on hematology and biochemistry parameters of blood in lactating goats (Šalavardić et al., 2021), while inclusion at 22% or greater negatively increased blood urea nitrogen (BUN) and creatine kinase in goats (Abrahamsen et al. 2021b). An increase in blood urea nitrogen was also observed in heifers fed HSC (Winders et al. 2022b). Feeding SHB in lactating dairy cows or lambs did not affect blood urea nitrogen (Irawan et al. 2024; Parker et al. 2022).

A recent study conducted in Germany reported a decrease in respiratory and heart rate in lactating dairy cows fed hemp silage (Wagner et al. 2022). Although not directly measured, the study also reported some negative effects on the behavior of the animals, such as increased salivation and tongue play and abnormal posture appearance. Researchers from Kansas, USA, reported instead a positive response of Holstein steers to feeding flower hemp, as indicated by the favorable decrease of some oxidative stress and inflammation biomarkers and a longer lying time (Kleinhenz et al. 2022). Our study with dairy cows revealed a possible increase in oxidative stress and inflammation in cows fed SHB, particularly due to an increase in ROM and ceruloplasmin in plasma (Irawan et al. 2024). In the same study, we did not observe any effect on animal behavior. The discrepancies from the studies could be explained by the form of hemp offered and the type of animals, but, as previously argued (Irawan et al. 2024), the level of cannabinoids ingested by the animals cannot explain those data, as it was larger in our study compared to the other studies. Overall, the data produced so far indicates a low risk of health issues associated with feeding hemp and its byproducts to ruminants.

#### Rumen metabolism and microbiome

Considering that hemp byproducts contain various phytochemicals and distinct nutritional properties, especially the high proportion of PUFA and the high protein content, investigating their role in rumen microbiota is highly relevant. There is a scarcity of studies investigating how hemp byproducts may influence rumen metabolism and the microbiota residing within it. Quantitative summary from 4 studies via meta-analytical approach indicated that hemp byproducts did not impact the total production of volatile fatty acids (VFA) or the proportion of acetate and propionate in the rumen (Supplementary Table 1). However, the proportion of butyrate was found to decrease (SMD = −1.098; *P* = 0.017) by feeding hemp byproducts. In our studies, the levels of β-hydroxybutyrate in the blood of lambs were influenced by the consumption of SHB (Parker et al. 2022) or after withdrawal of SHB from the diet in dairy cows (Irawan et al. 2024). Production of VFA, particularly butyrate, linearly decreased by feeding SHB to dairy cows (Wang et al. 2023) without altering the molar proportions of VFA or the concentration of β-hydroxybutyrate in blood. Feeding increased doses of HSB in goats decreased linearly the production of all rumen VFA (Abrahamsen et al. 2021b). In another study, feeding HSB to steers led to increased production of VFA and ammonia in the rumen. This was accompanied by higher concentrations of isobutyric acid but lower concentrations of isovaleric acid, with no significant changes observed in other VFA (Winders et al. 2023). Overall, hemp byproducts have a different effect on VFA production, with HSB having a positive effect on beef cattle but not in goats and SHB having a negative effect in dairy cows.

Despite a minor effect on the proportion of various VFA, rumen microbial rRNA 16S sequencing revealed alteration of several microbial communities in the rumen, with enrichment of bacteria that are negatively associated with feed efficiency, as a consequence of feeding HSB to steers (Winders et al. 2022a). In lactating cows that were fed SHB, only minor changes in the rumen and fecal bacteria were observed (Wang et al. 2023), including a decrease in the proportion of *Bacteroidota* and *Prevotellaceae* in cows fed 6% SHB (but not in cows fed 11% SHB). Similarly, in our trial, feeding either 10% or 20% SHB in lambs had minor effects on the rumen microbiota, with only a significant change in beta diversity of rumen microbiota by feeding 20% SHB whereas 10 and 20% SHB inclusion decreased the relative abundance of Bacteroides and several other phyla including *Chloroflexi* *,* *Campilobacterota, Cyanobacteria, Actinobacteria* *,* *Verrucomicrobiota*, and *Proteobacteria* (Irawan et al. 2024 unpublished data). In ruminants, a higher proportion of Bacteroides is associated with improved feed efficiency (Li et al. 2019). A lower feed efficiency was observed when HSB was included in the diets, as reported in several studies (Abrahamsen et al., 2021; Winders et al. 2022a). However, higher feed efficiency was observed in animals fed SHB (Parker et al. 2022; Wang et al. 2023).

### Global regulatory environment for hemp byproducts as animal feed

Regulations are never static and constantly evolving. This section aims to provide the most recent updates on hemp regulation as animal feed. These are relevant at the time of publication until new statutes and rules are promulgated. The regulation of hemp farm products and feed ingredients is a combination of historical use, political nuance over 100 years of drug policy, and growing demand for innovative agriculture and manufacturing products. When considering the additional regulation of food and feed, the landscape is filled with grey areas and the need for continued investigation, to make up for decades of lost research opportunities. Reviewing regulation and focusing on commonalities provides clarity when examining the jurisdictions that include animal feed in hemp definitions, those that limit grain or consumption of hemp as food, and those that are working through the process of gaining new feed approvals for industrial hemp through arduous federal regulations.

#### Hemp as an agricultural commodity

In countries where prohibition of hemp cultivation never occurred, there is little need for regulation of seed byproducts in animal feed. In those countries, enough regulatory confidence exists that animal feed is included in its definition. The global regulatory environment of hemp as an agricultural commodity in different regions is provided below:

- Central and Southeast Europe: Serbia includes the production of seeds for animal nutrition in its rulemaking, and in Bosnia and Herzegovina hemp is allowed for fiber and animal feed seed production (LLC 2023). Bulgaria succinctly allows fibers and seeds for animal feed or sowing only. Similarly, Croatia includes the production of seeds for animal feed and reproduction (LLC 2023). In Romania, there are several active industrial hemp processors located near production sites, which market hemp seeds for humans and animals; however, the definition does not specify industrial hemp’s legal status as animal feed in its rulemaking (USDA-GAIN 2020a). Poland also lacks clear regulatory guidelines, while broadly including hemp or hemp-derived products in its definition, with no restrictions on processing and permits for hemp to be used for fiber, food, cosmetics, pharmaceutical products, and building materials (LLC 2023).
  Slovenia permits the use of seeds, leaves, and stems of industrial cannabis for food and industrial purposes. In describing the regulation of hemp production, veterinary purposes are mentioned along with medical and scientific research purposes (LLC 2023). The lack of clarity on animal feed is often exacerbated by a focus on controlling narcotics and emphasizes the regulation of hemp only after the enforcement of drug trafficking laws. In Slovenia, hemp is grown under licenses issued by its Ministry of Agriculture, but veterinary products are regulated under the Act on Production and Trafficking in Illicit Drugs.
- North and West Europe: The hemp industry in the Netherlands is responsible for 5% of the European Union’s annual hemp production (European Commission 2019). It has a well-established supply chain and processors successfully export hemp products without clear legal approval. Dun Agro, a Dutch processor working across the hemp product supply chain, includes animal feed supplements from leaf and seed in its portfolio (USDA-GAIN 2020b). Netherlands, as well as Romania, are not the only countries where it is fair to say that acceptance is high, but regulations remain unclear. Rather than clear acceptance, Great Britain instead utilizes its definition of cannabis to exclude hemp products separated from the rest of the plant, including mature stalk, fiber production, and the seed (LLC 2023). Italy also has a long history of hemp production. In 2016, the government of Italy allowed the cultivation of hemp without a government license or authorization (LLC 2023). It is not surprising considering that in the 1940 s, Italy was the second-largest producer of hemp in the world after Russia (USDA-GAIN 2020b). There is unabashed enthusiasm for the future of hemp in its guidance of Law No. 242 of 2016 regulates hemp for the development of local supply chains that enhance research, and promotion of a sustainable and eco-friendly economy, a new hemp economy (LLC 2023). In 2017, the Italian Ministry of Health set limits on THC content in hemp seed products at 5 mg/kg for hempseed oil and 2 mg/kg for hempseed and hempseed flour and supplements derived from hemp without clarifying animal feed as one of its purposes.
- Non-European countries: Known for its history of cultivating hemp, in 2021, India issued a notification in its Food Safety and Standards Authority that amended its regulations clarifying that hemp seed, hempseed oil, and hempseed flour shall be sold as food or used as an ingredient in a food for sale (Government of India, 2021).

#### Influence of drug regulation

Because of the history of drug laws across the world that led to prohibition in many countries, the lack of a clear separation between drug cannabis and industrial hemp is most impacting global approval of hemp in animal feed, regardless of whether it is composed of seed or other plant material. The influence of drug policy on the regulation of hemp-fed animals is pronounced, especially when considering the desire to introduce hemp as a feed ingredient for animals or animal byproducts intended to enter the human food chain. Exacerbating this issue is the lack of research due to some form of prohibition in most countries. The first regulation of cannabis as a drug occurred at the 1912 Hague International Opium Convention (IOC) (UNODC 2018). After World War 1 and in response to increasing drug use, the IOC was included in the peace treaties furthering its adoption. In 1936, the first international criminalization of drug trafficking occurred with the Convention for the Suppression of the Illicit Traffic in Dangerous Drugs (UNODC 2018). Many countries began prohibition, including the US, shortly after its passage. This culminated in the creation of the foundational document that lists controlled substances, including cannabis: the 1961 Single Convention on Drugs. Two countries reveal a strong adherence to regulating industrial hemp solely as drug cannabis: Hong Kong and Japan.

- Hong Kong: Hong Kong focuses its regulations on CBD, classifying it as a dangerous substance prohibited for use under the Dangerous Drugs Ordinance passed in 2023. In Hong Kong, the strict enforcement of any products containing CBD, combined with a reliance on imports for 95% of its food supply (FDA 2019; LLC 2023), explains why hemp as animal feed is not a priority. Israel also defines cannabis as a dangerous drug but excludes the oil extracted from its seeds in its Dangerous Drugs Ordinance. Recent amendments allow for the cultivation of cannabis in Israel after decriminalization in 2019, and the Minister of Health is currently considering the exclusion of CBD as well.
- Japan: Japan is another country with a millennia long history of hemp cultivation disrupted by drug trafficking laws. Its cultivation, while allowed for fiber from stems, food from seeds or for research purposes, has greatly declined since the first World War after the first regulation of cannabis in 1947. The Cannabis Control Ordinance began as a negotiation to allow cannabis native to Japan to be cultivated to protect its farmers. This was quickly followed by the Cannabis Control Act in 1948 which bans importation of cannabis but excludes mature stalks. It further excludes cannabis seeds and products made from the seeds in its definition of cannabis (USDA-GAIN 2023a). The most significant production of cultivated hemp acres occurred in 1952, with a total of 5,000 hectares. Since then, there has been a steady decline in hemp production, and in 2016, just six hectares were licensed (LLC 2023). Current use of hemp seed in Japan has primarily been for bird seed, accounting for around 80% of imports and only 20% for human consumption. Limited domestic production has created a reliance on imported hemp products, and there is not much expectation that a domestic Japanese hemp seed or hempseed oil industry will be developed in the future, leaving little motivation to address the legality or regulation of animal feed.

#### Operating in a grey area

Some countries that have been growing hemp for decades have only recently begun to regulate industrial hemp products.

- China: China has one of the longest relationships with hemp and has been cultivating it for thousands of years for rope and canvas. It was also the birthplace of the opium industry, leading to the IOC (UNODC 2018). The inclusion of cannabis in the IOC began global restrictions on cannabis and impacted the broad use and production of industrial hemp. This is evident in federal Chinese anti-drug laws prohibiting the cultivation of cannabis. But local action affects acceptance. Two provinces have promulgated regulations for industrial hemp, Yunnan in 2010 and Heilongjiang in 2017 (LLC 2023). The regulations in Heilongjiang define industrial hemp for industrial use, which include manufacturing building materials, fiber, food, medicines, and animal feed. Standards are being developed for industrial hemp seeds by China’s Ministry of Agriculture and Rural Affairs, including the allowable THC content (LLC 2023). One year later in 2019, China’s National Narcotics Control Commission repeated this clarification by confirming that cultivating and processing industrial hemp should be limited to fiber and seeds, excluding the medical and food additives uses (LLC 2023). However, there exists little clarity or regulation allowing hemp to be fed to animals.
- Russia: While Italy was a global hemp powerhouse in the 1940 s, hemp represented Russia’s most significant export leading the world in hemp cultivation. Most European hemp for sails and rope came from Russia, including 90% of hemp imported by Great Britain at the end of the eighteenth century (LLC 2023). But they did not escape the global war on drugs. In 1998, The Federal Law on Narcotics and Psychotropic Substances was passed but carved out a broad exception for specific varieties of plants containing narcotic substances that can be cultivated for industrial purposes (Russian Federation 2020). A decade later in 2007, the government further protected its industrial hemp legacy by legalizing hemp cultivation, which allowed limited production of hemp and specified the types of seeds that can be grown (LLC 2023). Given the history of hemp for fiber and textile production combined with little reference to seed or food uses, it is safe to assume that the focus of industrial purposes in Russia would be on the production of hemp fibers. Little mention of hemp as food and none as animal feed was observed. Instead, the regulatory focus is on the prevention of the cultivation of hemp for narcotic and psychotropic substances. Veterinary purposes are allowed in the Law on Narcotics and Psychotropic Substances, passed in June of 2019, which added to medical and veterinary purposes when using hemp containing dry masses of leaves and inflorescence of the uppermost flowering parts (Russian Federation 2020). This left the focus on fiber and medicinal applications rather than nutrition.

#### Establishing global regulations

**Fig. 9:**
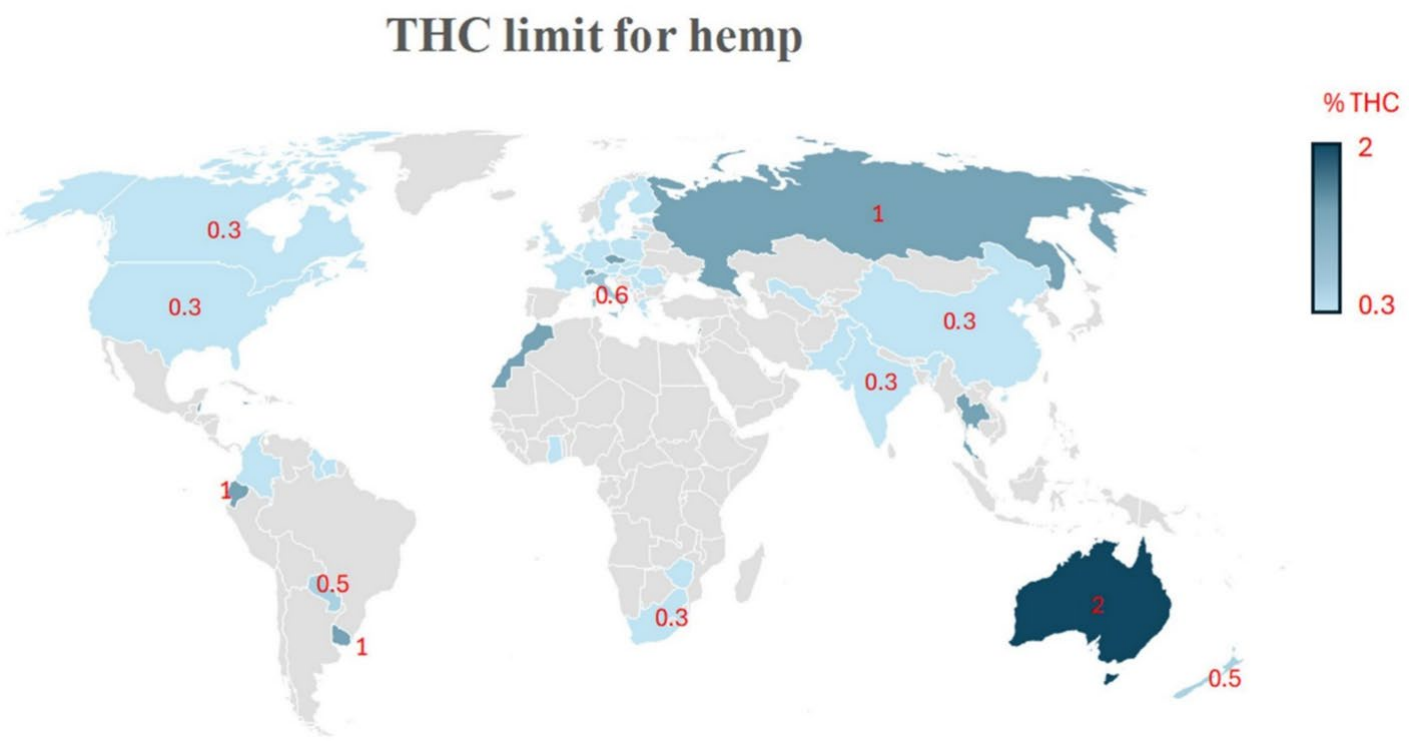
Limit of THC in hemp in various countries around the world (data are from Table 1 in FIHO 2024)

This section is devoted to countries with well-established feed approval processes actively seeking agency approval in 2024. It is important to remember that the war on drugs has slowed the approval process in several ways. Global prohibition of hemp cultivation prevented research and feeding trials, development of analytical methods needed to address THC content, and cannabinoid transference in animals and resulting byproducts. Additionally, new concerns over semi-synthetic THC isomers have reinforced the desire to push THC limits often lower than the ability of regulators to measure the concentration (Runco et al. 2016). A map of the legal limit of THC in industrial hemp is provided in Fig. 9 (FIHO 2024).

In many countries actively pursuing approvals of using hemp as a feed ingredient, the approval of cannabinoids as drugs for animals is further along. The development of new analytical methods has been recently reported in hemp byproducts and animal tissues (Chakrabarty et al. 2022; Smith et al. 2023), which helps to establish the standard method for cannabinoid quantification in future animal model studies. However, challenges remain with regulatory agencies and jurisdictions charged with enforcement, especially concerning validating methods to detect trace amounts of cannabinoids found in seed products. It is worth noting that other hemp feedstuffs have been investigated, including forage, silage, and post-extracted biomass where cannabinoid concentrations are higher and more straightforward to quantify.

Several countries are leading the development of feed ingredient definitions, safety standards, and action limits for compliance, and most have established guidelines and processes for feed approvals (Table 5). Development of these processes has taken years of legislation, rulemaking, and engagement with university researchers and industry leaders. Although hemp seed was a common animal feed, the US was also impacted by international drug laws and passed the Marihuana Tax Act in 1937. It was not until 2018 that hemp was again legal to cultivate across the United States for agricultural purposes (Johnson 2019).

**Table 5:** Regulations of permitted concentration of total THC contaminants in food for human consumption in various countries

Hemp byproducts	Global Hemp Compliance, Food and Animal Feed Limits (mg/kg or ppm, unless indicated otherwise)								
	USA	US Feed	EU	EU Feed	Canada	Germany	Australia	New Zealand	China
Compliance^a^	0.3%		0.2%		0.3%	0.3%	1.0%	0.35–0.50%	0.3%
Whole seed	10		5	5 (3)	10				
Dehulled seed			2.5						
Seed oil	4		10	15 (7.5)	10			Permitted^b^	
Seed meal		2 (10)		5 (3)	10				
Seed protein			3.5		10				
Complete feed				3 (0.5)					

Values in parentheses are industry recommendations

^a^Percent dry weight

^b^Permitted for human consumption

- United States of America:The first laws regulating food and feed were passed in 1906 in the US Food and Drug Act (FDA 2019). The law focused on product labeling and prohibited the addition of ingredients that would substitute for the food, conceal damage, pose a health hazard, or constitute a filthy or decomposed substance (FDA 2019). However, that changed with the passage of the 1938 Federal Food, Drug, and Cosmetic Act (FD&C) (Lam and Patel 2023). The FD&C created the FDA, and the pre-market approval process was born (Lam and Patel 2023). Because the Marihuana Tax Act criminalized cannabis cultivation, there was no need to accept hemp into traditional agriculture commodities and common uses like feed. The resulting pre-market approval requires extensive safety trials and end-product testing for the ingredient, feed, and final animal byproduct. When the research is compiled into either a Generally Recognized as Safe (GRAS) or a New Feed Additive Petition, it is the FDA-CVM that receives the application and grants acceptance. For international purposes and the focus of this review, agency acceptance is the goal.
  Currently, in the US, the first submission for hempseed meal to be fed to laying hens has been accepted by the FDA-CVM (Hemp Feed Coalition 2024) and awaits approval by the state-led feed regulatory body of AAFCO (2018). Upon approval, the definition of the ingredient, including its label, guaranteed analysis of nutrients, inclusion rate, use, and animal category, is published in the AAFCO Official Publication for state regulators who enforce feed at the state level. Compliance of crops and feed is most often conducted by State Departments of Agriculture overseen by the USDA. Based on FDA rules, the USDA is quite clear on prohibiting products with added THC and CBD for interstate commerce and turns again to the agency for pre-market approval of hemp feed ingredients. The submission of the application is usually made by a commodity group or private industry that can afford the expense and wait four years on average for a single ingredient, single species approval. While the researchers work to close scientific and analytical gaps, hemp farmers and processors are operating and meeting industry standards. In the US, there is a focus on THC and confusion over cannabis as well as new synthetic THC analogs such as Δ^8^-THC. Despite historic use and the US acceptance of a GRAS notice for human consumption, limits below what industry standards are being proposed by the FDA-CVM, including a limit for CBD, something that has not occurred at the regulatory level of animal feed.
- Canada: North of the US, Canada has a 10-year head start and developed a strong hempseed market when they legalized the cultivation and processing of industrial hemp in 1998 (Canada 2024a; Canada 2024b). As part of the Cannabis Act of 2018, the first regulations were promulgated in the Industrial Hemp Regulations (IHR) (Canada 2024b), part of the Cannabis Act of 2018. Five years later, in 2022, Canada’s global export of hemp seed products was worth $53.6 million, with 95% exported to the US (USDA-GAIN 2023b). Domestically, the regulation of hemp containing products with less than 10 μg/g THC are exempt from the IHR and the Cannabis Act licensing rules (LLC 2023), and hemp seeds are excluded from the Cannabis Act. The Canadian Food Inspection Agency (CFIA) is charged with regulating food and feed. Currently, there are no hemp livestock feed ingredients approved, and each hemp product intended for use as a feed or feed ingredient requires a separate approval. Fortunately, the CFIA has been working with industry leaders and researchers to develop guidance, including the document Regulation of Hemp Products for Use in Livestock Feeds (Canada 2024a; Canada 2017). Even with their engagement, it has been a long process stretching over five years. However, approval is expected in the next two years for hempseed meal, oil, and other ingredients to be fed to a few livestock species.
  Like many other countries, in Canada, veterinary products, including hemp, have a regulatory pathway through a voluntary registry of Veterinary Health Products (**VHP**) (Canada, 2017). This program oversees the sale and import of veterinary products with a voluntary registry. The ingredients enlisted as VHP are described as low-risk drugs in dosage form that promote the health and welfare of both pets and livestock (Canada, 2024b). These drugs include vitamins, minerals, and traditional medicines that have historical use. The program allowed compliance and enforcement for manufacturing, labeling, and quality products intended for animal use when it took effect in 2017. The list of current VHP includes CBD supplements and other hemp-containing products, due to lack of federal approval.
- European Union: Europe is considered the world’s largest hemp-producing market, with many countries lifting their ban on hemp cultivation in the 1990 s. France is driving the hemp industry, producing 70% of European hemp, followed by the Netherlands and Austria (European Commission 2019). Including hemp in its New Green Deal and standard agriculture policy has led to a significant increase in hemp production of 62.4% since 2015 (LLC 2023).
  The European relationship with industrial hemp is still evolving despite the long history of its cultivation. Sweden is an excellent example of this, with evidence of its farming dating to the Iron Age 550 BC-1150 AD (Hessle et al. 2008) and its expansion through the Second World War, with over 2000 hectares grown in 1942. It was not until 1965 when the cultivation of hemp was prohibited because it contained the narcotic substance tetra-hydro-cannabinol (THC) (Hessle et al. 2008) that Sweden stopped its production. In 2003, the Swedish Board of Agriculture allowed the cultivation of hemp that contains less than 0.2% THC. The current regulation and approval of industrial hemp as animal feed falls under the jurisdiction of the European Union, which in 2024 raised the THC limit to 0.3% THC, aligning with the USDA limits.
  The process to gain approval is much the same in both Canada and the US, with the submission of an application by the company wishing to manufacture and market the feed product. The application must undergo agency scrutiny by the European Commission and the approval process is well documented in the regulation of novel foods (The European Parliament and the Council of the European Union 2015). Once approved, regulation of feed culminates in a publication. The Catalogue of Feed Materials and inclusion in the online Feed Material Register (EU Feed Chain Task Force 2024) allow use in the denominations within the EU. The registry currently lists seven hemp and hemp byproducts, including hempseed husks, hemp components, cold-pressed hemp oil, hemp meal, hemp extraction meal (post-extracted biomass), and hemp shells, provided that the materials are below the EU limit of 0.2% THC for industrial hemp. A differentiation of products that include hemp extracts, isolated CBD, and other cannabinoids are considered feed additives, and to date, no company has undergone premarket authorization. While rules for hemp feed product approvals in the EU have been promulgated by industry and agencies working together, it is not surprising that the establishment of maximum levels of THC in feed materials remains a concern. The European Industrial Hemp Association has proposed limits that mimic food products and hopes for their adoption in 2025.
- New Zealand and Australia:On the other side of the world, New Zealand and Australia have burgeoning hemp industries, but well-established processes and rules for approving food and feed ingredients. New Zealand classifies cannabis as a controlled substance in the Misuse of Drugs Act established in 1975, and licensing for cultivation is still strictly controlled (Elias 2006). The addition of hemp regulations to the Misuse of Drugs Act in 2006 allowed for the licensing and cultivation of industrial hemp and was further amended in 2018 to adopt changes to the Australia New Zealand Food Standards Code to allow the possession, use, trade, and import and export of low THC hemp products (Elias 2006). However, it does not include animal feed. The government has dedicated resources to clarify and correct public confusion with a press release stating recent media comments that it is legal to use unprocessed harvested hemp, or hemp products such as baleage or silage, as an animal feed in New Zealand are incorrect (Ministry of Primary Industries 2018).
  Regulation of feed and veterinary products occurs under the Agricultural Compounds and Veterinary Medicines Act (ACVM) of 1997. The ACVM provides a pathway for authorization of agricultural compounds and clarifies that “there are currently no hemp or hemp products for animal feed registered[...] and the exemption status of these products has not been confirmed.” However, hemp industry associations are working to establish an exemption for hemp-based nutritional products for animals if they have no detectable THC or CBD and meet all the requirements for exemption under the ACVM Act. New Zealand has dedicated resources to establishing hemp food in its food supply chain and published “The Guide to Hemp Seeds as Food''in 2022. There is optimism that the exemption will occur and open a path to legally feeding hemp seeds and their by-products to animals.
  Australia and New Zealand share a Food Standards Code which includes hemp as a food product and provides clarity for human consumption, although most regulation occurs at the local, state, and territory levels. While Australia legalized hemp cultivation in 1967 under the Narcotic Drugs Act, it did not provide licensing structures or a framework for cultivation or production (LLC 2023). This combination left a vague federal picture of the legality of cultivation and the production of hemp products, including those for animals, instead of charging the states with rulemaking. However, there is enthusiasm in Australia for hemp applications in textiles, building materials, paper, rope, and animal feedstock. The federal government gave the industry good reason to feel optimistic. In its update of the Australia New Zealand Food Standards (FSANZ 2017) Code, they included food derived from the seeds of low Δ^9^-THC varieties of Cannabis sativa (low THC hemp) (FSANZ 2017). Together, the two countries are engaged in animal feed trials using hemp seed products. They are especially interested in identifying a new feedstock for their cattle industry. Still, at the time of this writing, there is no legal approval for hemp feed in either New Zealand or Australia.
- Colombia:Colombia is an intriguing addition to this section, where the adoption and use of industrial hemp have been promoted as a mechanism to provide opportunities and jobs outside of illicit markets. The passage of the Key Regulations in 2022 created a legal framework for the cultivation and use of hemp fiber and grain. Not only did it remove non-psychoactive cannabis or hemp from its list of controlled substances, but it also allowed for the commercialization, import, export, and transportation of grain and plant components for industrial purposes. The law further clarifies that industrial purposes, including products for animal use or consumption, may only be manufactured from grain or hemp plant components if they comply with the Columbian Agriculture Institute regulations (LLC 2023). The regulation of hemp products for human and animal consumption, including food, beverages, cosmetics, and supplements with a limit on THC, is regulated by the Ministry of Health and Social Protection.

## Conclusions and future thrust

The data generated so far suggests that hemp byproducts are safe as feed ingredients for most ruminant species. However, only hempseed meal is legalized as feed ingredients and only in a few countries, while SHB has not been legalized yet. The legal status of SHB makes research on live animals challenging. In the US, the animals exposed to SHB cannot enter the food chain, according to the FDA. It is a requirement that animals used in those experiments be euthanized and landfilled at the end of the experiments. On the one hand, this can provide the opportunity to collect more samples for analyses, helping to generate more solid data to submit to FDA-CVM or other government agencies of other countries for possible approval status; on the other hand, it increases the cost of research. In the US, it is possible to obtain a Food Use Authorization from the FDA-CVM to keep the animals after the experiment, as we did in our experiment with dairy cows (Irawan et al. 2024).

The critical aspect of legalization is the solid evidence that biomass or other byproducts will not introduce psychoactive compounds such as Δ^9^-THC and CBD to consumers. Hence, quantifying the residuals of cannabinoids in meat and milk using validated and robust analytical methods should become the top priority of the research agenda. Therefore, there is an urgent need to develop analytical methods that can rapidly and efficiently quantify trace amounts of cannabinoid compounds in various biological matrices from livestock-fed hemp byproducts. For this purpose, available methods commonly used in the forensic area can be modified and re-developed. In addition, it is also promising to assess the use of other byproducts or the available biomass for other purposes due to the antimicrobial properties of hemp, such as curing mastitis or microbial diseases in livestock. The Federation of International Hemp Organizations compiled current regulations and approvals in a white paper in 2024 (FIHO 2024). While this paper is unpublished, it is the most comprehensive data collection on hemp feed approvals to date. The data was gathered through self-reporting from the countries listed. The white paper highlighted the major limitations in creating a global feed market for hemp and the legalization of its byproducts as feed ingredients. Those include the influence of anti-drug laws, the creation of new feed regulatory agencies, the need for safety studies focused on the transference of THC and CBD, and regulators asking for thresholds of THC in byproducts that are below most established contaminants of concern and/or below therapeutic or pharmaceutical applications.

There is no question that hemp grain and potentially other hemp products will eventually be approved as animal feed ingredients. Still, it remains unclear what country, company, or industry association will be the first international exporter. What is clear is that global leadership is needed to develop both markets and the research needed to make hemp as animal feed a reality.

## Supplementary Information

Supplementary Material 1.

Supplementary Material 2.

## Data Availability

Data is provided within the manuscript or supplementary information files.

## Abbreviations

AAFCO: Association of American Feed Control Officials
ACVM: Agricultural Compounds and Veterinary Medicines Act
ADF: Acid detergent fiber
ADG: Average daily gain
CBC: Cannabichromene
CBD: Cannabidiol
CBDA: Cannabidiolic acid
CBE: Cannabielsoin
CBG: Cannabigerol
CBL: Cannabicyclol
CBN: Cannabinol
CBGA: Cannabigerolic acid
CFIA: Canadian Food Inspection Agency
CP: Crude protein
DDGS: Distillers dried grains with soluble
DHA: Docosahexaenoic acid
DPPH: 2,2-Diphenyl-1-picrylhydrazyl
EAE: Enzyme-assisted extraction
FA: Fatty acids
FDA-CVM: Federal Drug Administration Center for Veterinary Medicine of the USA
GRAS: Generally recognized as safe
HSB: Hempseed byproducts (hempseed cake or hempseed meal)
HSO: Hempseed oil
IHR: Industrial Hemp Regulations
MAE: Microwave-assisted extraction
NDF: Neutral detergent fiber
PLE: Pressurized liquid–liquid extraction
PUFA: Polyunsaturated fatty acids
SFE: Supercritical fluid extraction
SBM: Soybean meal
SHB: Spent hemp biomass
THC: Tetrahydrocannabinol
VFA: Volatile fatty acids
VHP: Veterinary Health Products
VOC: Volatile organic compounds
