# Supplementary material for: Use of industrial hemp byproducts in ruminants: a review of the nutritional profile, animal response, constraints, and global regulatory environment
Source: J Cannabis Res. 2025 May 14;7:25. doi: 10.1186/s42238-025-00279-7 (PMC12076842; doi:10.1186/s42238-025-00279-7)
Supplement: Supplementary file 2 — Supplementary Material 2. [file 42238_2025_279_MOESM2_ESM.docx]

# Supplementary Materials

# *Meta-analysis approaches*

**Literature search*.*** Articles were searched using keywords including “hemp,” “cannabis sativa,” “feed,” “dairy cows,” “beef,” “lambs,” “sheep,” “goat,” and “ruminant” in Web of Science, Scopus, Science Direct, PubMed, and Google Scholar. This search was done to generate studies published by January 2024. All titles generated from each search were imported, and duplicate titles were removed before further selection was made to identify eligible studies. No limitation on publication years was applied.

**Inclusion criteria*.*** The following inclusion criteria were applied: (a) peer-reviewed articles; (b) *in vivo* studies that assessed the impact of hemp or hemp byproducts as feed ingredient for ruminant livestock; (c) included a comparison of control and intervention groups; (d) provided sufficient information about the methods including study design, randomization, replication, intervention, data acquisition and measurement, and data analysis; and (e) reported the study outcome along with their variance (standard deviation and/or standard error). The following outcomes were included in the dataset: production performance, nutrient intake, nutrient digestibility, rumen fermentation, fatty acids profile (milk and/or meat), and carcass and meat quality.

**Dataset construction*.*** A total of 20 studies were deemed eligible for the meta-analysis (**Table 4**) after being selected according to our criteria. Data from each study, including the information of authors, country, details of animals (type, age, parity, breed), number of replicates, study design, types of diets, types of hemp, inclusion levels (% DM), and outcome data with their variance were input in the spreadsheet. The standard error (SE) was used in the final dataset, and it was calculated from standard deviation (SD) when SE was not reported, where SE = SD/sqrt(n), where n = sample size or the number of replicates. Graphical data were extracted using the online tool WebPlotDigitizer (<https://apps.automeris.io/wpd/>).

**Data analysis*.*** Publication bias was not calculated in this meta-analysis due to a relatively low number of studies. We considered all studies included in this analysis scientifically sound after scrutinizing the paper’s quality, and therefore, no strict sensitivity, risk of bias, or other assessments that are typical in meta-analysis reviews were applied. The meta-analysis was performed in the R studio (RStudio version 4.3.0) using the “meta” package (Schwarzer et al., 2015). Between-study heterogeneity and significance of heterogeneity were calculated using *I^2^* and Cochran’s *Q* statistic (Higgins et al., 2003) with the DerSimonian-Laird estimator to explain the total variance (%) across studies. The *I^2^* was classified as no evidence (0< *I^2^* ≤ 25%), low (25%< *I^2^* ≤ 50%), moderate (50%< *I^2^* ≤ 75%), and high (*I^2^* ≥ 75%) heterogeneity (Higgins et al., 2003). Our results indicated an *I^2^* ≥ 75%, suggesting high heterogeneity between studies. It is suggested that a random effect model (REM) meta-analysis is appropriate to estimate the standardized means difference (SMD) when high heterogeneity is observed (Higgins et al., 2003). By default, the Hedges' *g* effect size and its variance were used to estimate the SMD, weighted by the inverse matrix from the studies using the following equations: $SMD= \frac{\mu_{1}- \mu_{2}}{SP}SP$ (equation 1)

Where *SP* = ${\sqrt{\frac{\left( n_{1}-1 \right){SD}_{1}^{2}+\left( n_{2}-1 \right){SD}_{2}^{2}}{n_{1}+n_{1}-2}}}$ (equation 2)

and inverse-variance weighted = $\frac{\sum Yi(1/{SE}_{i}^{2})}{\sum(\frac{1}{{SE}_{i}^{2}})}$ (equation 3)

where μ_1_ = mean of the dietary intervention group, μ_2_ = mean of the control group, SP = combined SE, SE_1_ = SE of the dietary intervention group, SE_2_ = SE of the control group, n_1_ = the sample size of the dietary intervention group, n_2_ = the sample size of the control group, Yi = the estimates of the intervention effect in the study, SE = the standard error of the estimate (Schwarzer et al., 2015). The SMD was presented as a 95% confidence interval (95% CI). Furthermore, a subgroup meta-analysis was conducted to examine the effects of the hemp byproducts in each different type of animal (dairy cows, beef cattle, and small ruminants).

Excluded (n = )

- Non-research articles: 50
- Duplicate articles and non-related title: 176

Articles were not related to the ruminant feeding studies or did not contain outcome of interest (n = 19)

No study was excluded

Article outputs using a combination of keywords “Hemp”, “Cannabis sativa”, “Dairy cows”, “Beef”, “Lamb”, “Goat” Scopus (n = 118), Web of Science (n= 153)

Number of studies used for meta-analysis (n= 26)

Studies left for final full-text evaluations (n= 26)

45 titles were screened in MS. excel and selected titles were downloaded

**Identification**

**Screening**

**Eligibility**

**Inclusion**

**Figure 1**. Flowchart of article selection

**Supplementary Table 1**. Outputs of meta-analysis results of all included parameters

| Subgroups | *N* | Estimate (RSM, 95% CI) | | | τ | *P*-value | Heterogeneity | |
| --- | --- | --- | --- | --- | --- | --- | --- | --- |
|  |  | SMD | Lower | Upper |  |  | *I^2^* | *Q* |
| DMI |  |  |  |  |  |  |  |  |
| Overall | 37 | -0.021 | -0.634 | 0.592 | 1.787 | 0.945 | 86.8 | <0.001 |
| Small ruminants | 15 | -0.697 | -1.438 | 0.043 | 0.338 | 0.065 | 81.0 |  |
| Dairy cows | 18 | 0.215 | -0.767 | 1.198 | 0.501 | 0.668 | 90.0 |  |
| Beef steers | 4 | 1.661 | -0.087 | 3.409 | 0.892 | 0.062 | 51.0 |  |
| HP | 6 | -2.210 | -3.301 | -1.120 | 0.556 | <0.001 | 28.5 |  |
| SHB | 5 | -1.139 | -2.777 | 0.499 | 0.836 | 0.173 | 88.0 |  |
| HSB | 26 | 0.631 | -0.005 | 1.266 | 0.324 | 0.052 | 78.0 |  |
| ADG |  |  |  |  |  |  |  |  |
| Overall | 20 | -0.638 | -1.688 | 0.412 | 2.305 | 0.234 | 89.4 | <0.001 |
| Beef cattle | 2 | -1.805 | -3.397 | -0.214 | 0.812 | 0.026 | 0.00 |  |
| Dairy cows | 3 | 0.208 | -0.631 | 1.047 | 0.428 | 0.629 | 83.0 |  |
| Small ruminants | 16 | -0.880 | -2.290 | 0.530 | 0.719 | 0.221 | 91.0 |  |
| SHB | 4 | -0.182 | -2.001 | 1.637 | 0.928 | 0.844 | 66.0 |  |
| HSB | 15 | -1.128 | -2.409 | 0.152 | 0.653 | 0.084 | 86.0 |  |
| Feed Efficiency |  |  |  |  |  |  |  |  |
| Overall | 20 | -1.351 | -2.366 | -0.337 | 2.165 | 0.009 | 85.5 | <0.001 |
| Small ruminants | 10 | -0.421 | -0.802 | -0.040 | 0.194 | 0.030 | 0.00 |  |
| Dairy cows | 8 | -0.777 | -2.665 | 1.110 | 0.963 | 0.419 | 95.0 |  |
| Beef cattle | 2 | 0.397 | -0.418 | 1.286 | 0.452 | 0.379 | 0.95 |  |
| SHB | 2 | 1.394 | 0.643 | 2.146 | 0.383 | <0.001 | 0.00 |  |
| HSB | 14 | -0.957 | -1.927 | 0.013 | 0.494 | 0.053 | 89.0 |  |
| Hot carcass weight | 14 | -0.229 | -0.751 | 0.292 | 0.808 | 0.389 | 64.1 | 0.001 |
| Dressing, % | 13 | -0.442 | -1.320 | 0.436 | 1.494 | 0.324 | 87.2 | <0.001 |
| Milk yield, kg/d |  |  |  |  |  |  |  |  |
| Overall | 13 | 1.024 | -0.124 | 2.171 | 1.991 | 0.080 | 88.2 | <0.001 |
| HP | 3 | -1.217 | -2.260 | -0.175 | 0.531 | 0.022 | 0.00 |  |
| SHB | 3 | 0.774 | 0.016 | 1.532 | 0.387 | 0.045 | 65.0 |  |
| HSB | 7 | 2.111 | 0.507 | 3.713 | 0.818 | 0.009 | 86.0 |  |
| Milk Fat, % | 18 | -0.089 | -1.502 | 1.323 | 2.964 | 0.902 | 92.7 | <0.001 |
| Milk Protein, % | 16 | -0.357 | -0.883 | 0.169 | 0.568 | 0.184 | 81.8 | 0.0005 |
| Milk Lactose, % | 16 | -0.322 | -0.955 | 0.311 | 1.154 | 0.319 | 86.1 | <0.001 |
| DM Digestibility | 14 | -2.440 | -7.008 | 2.128 | 8.545 | 0.295 | 96.1 | <0.001 |
| NDF Digestibility | 8 | -1.492 | -2.894 | -0.091 | 1.844 | 0.037 | 92.0 | <0.001 |
| Total VFA | 9 | -0.200 | -2.106 | 1.705 | 2.799 | 0.837 | 95.1 | <0.001 |
| Acetate, % | 9 | -0.628 | -2.378 | 1.122 | 2.537 | 0.482 | 94.5 | <0.001 |
| Propionate, % | 9 | -1.022 | -2.762 | 0.718 | 2.492 | 0.249 | 89.9 | <0.001 |
| Butyrate, % | 9 | -1.098 | -1.996 | -0.199 | 1.170 | 0.017 | 73.7 | 0.002 |
| Fatty Acid Profile |  |  |  |  |  |  |  |  |
| C18:2 n6, % | 10 | 1.323 | 0.126 | 2.520 | 1.826 | 0.031 | 89.3 | <0.001 |
| C18:3 n3, % | 13 | 5.450 | 1.823 | 9.138 | 6.529 | 0.003 | 89.7 | <0.001 |
| n6, % | 10 | 1.589 | 0.363 | 2.815 | 1.875 | 0.011 | 90.5 | <0.001 |
| Milk (SHB) | 3 | 1.034 | -0.114 | 2.181 | 0.585 | 0.077 | 68.0 |  |
| Meat (HSB) | 6 | 1.629 | -0.322 | 3.581 | 0.996 | 0.102 | 94.0 |  |
| n3, % | 12 | 2.181 | 0.787 | 3.574 | 2.023 | 0.002 | 84.6 | <0.001 |
| Milk (SHB) | 5 | -0.207 | -1.603 | 1.187 | 0.712 | 0.770 | 81.0 |  |
| Meat (HSB) | 7 | 3.510 | 2.589 | 4.430 | 0.469 | <0.001 | 55.0 |  |
| n6/n3 | 13 | -0.073 | -2.686 | 2.539 | 4.698 | 0.956 | 91.3 | <0.001 |
| Milk (SHB) | 3 | 4.771 | -2.909 | 12.45 | 3.918 | 0.223 | 90.0 |  |
| Meat (HSB) | 9 | -1.350 | -4.173 | 1.472 | 1.440 | 0.348 | 93.0 |  |

N = number of comparisons; SMD = standardized mean difference (estimated based on random effect models or RSM, adjusted by Hedges’ g effect size and presented at 95% confidence interval); τ = variance; *I^2^* = heterogeneity (%), Q = significance of the within-studies heterogeneity

HP = hemp plant (leaves and/or flower) containing cannabinoid; SHB = spent hemp biomass, an extracted byproduct from cannabidiol oil extraction; HSB = hempseed byproduct with no or very minimal cannabinoid
